# mGluR5 in EC^CCK^ to BLA Circuit Modulates Depressive‐Like Phenotypes through CCK Signaling

**DOI:** 10.1002/advs.202523115

**Published:** 2026-05-08

**Authors:** Muhammad Asim, Huajie Wang, Gao Qianqian, Abdul Waris, Jufang He

**Affiliations:** ^1^ Department of Neuroscience City University of Hong Kong Kowloon Tong Hong Kong China; ^2^ Centre For Regenerative Medicine and Health, Hong Kong Institute of Science and Innovation Chinese Academy of Sciences Hong Kong China; ^3^ Department of Psychiatry and Behavioral Sciences Stanford University Stanford California USA; ^4^ Research Centre for Treatments of Brain Disorders City University of Hong Kong Kowloon Tong Hong Kong China

**Keywords:** amygdala, cholecystokinin, depression, entorhinal cortex, mGluR5

## Abstract

Dysregulation of metabotropic glutamate receptor 5 (mGluR5) and cholecystokinin (CCK) signaling has been implicated in major depressive disorder (MDD), but the underlying circuit mechanisms remain unclear. Here, we define how mGluR5 regulates depressive‐like behaviors through CCK signaling in the entorhinal cortex (EC) to basolateral amygdala (BLA) pathway. Anatomical tracing and optogenetics show that CCK‐expressing neurons in the EC project to the BLA and that their activation increases glutamatergic activity in this region. Bidirectional circuit manipulation establishes causality: optogenetic stimulation induces, whereas inhibition alleviates, depressive‐like phenotypes. Expansion microscopy reveals postsynaptic mGluR5 enrichment along this pathway. Chronic social defeat stress (CSDS) downregulates mGluR5 in the BLA. Pharmacologically, mGluR5 antagonism phenocopies CCK‐driven pro‐depressive effects, whereas CCK knockout mice resist behavioral consequences of mGluR5 inhibition. Mechanistically, mGluR5 agonism suppresses CCK release, disrupts BLA long‐term potentiation, and mitigates CSDS‐induced behaviors. Circuit‐specific mGluR5 knockdown in the EC^CCK^→BLA pathway increases stress susceptibility. These findings identify an mGluR5‐CCK axis within an EC^CCK^→BLA circuit that governs stress‐induced affective states.

## Graphical abstract

Dysregulation of mGluR5 and CCK signaling contributes to major depressive disorder, yet circuit‐level mechanisms remain unclear. Here, the EC^CCK^→BLA pathway is identified as a critical regulator of affective behavior. mGluR5 modulates synaptic function and CCK signaling within this circuit, controlling stress susceptibility and depressive‐like states, and revealing a novel mechanism underlying mood regulation.

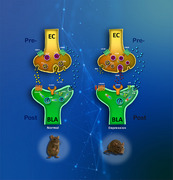

## 1 Introduction

Major depressive disorder (MDD) is a chronic, debilitating condition and a leading contributor to global disease burden [1, 2]. Affecting approximately 17% of the population over a lifetime, its incidence rose by over 25% during the COVID‐19 pandemic [3]. Chronic exposure to adverse experiences is a major risk factor for MDD and related affective disorders [4, 5], and such stressors are known to drive synaptic remodeling in limbic circuits involved in emotional processing [6, 7, 8, 9]. Among these, the basolateral amygdala (BLA) serves as a central hub for encoding affective valence and exhibits experience‐dependent synaptic plasticity in response to aversive stimuli. Recent work has demonstrated that activation of glutamatergic neurons in the BLA (BLA^Glu^) contributes to the expression of depressive‐like behaviors [10]. The BLA receives convergent sensory inputs across modalities [11, 12], but the upstream cortical drivers of BLA activity in the context of stress remain poorly defined.

The entorhinal cortex (EC), a major cortical input to the BLA [10, 13], is increasingly recognized as a key modulator of affective behavior. Beyond its well‐established role in spatial and contextual memory processing [14, 15], the EC has emerged as a key node linking sensory experience with emotional and motivational states. Indeed, EC projections to regions such as the visual cortex and dentate gyrus regulate neurogenesis and influence depressive‐like phenotypes [16, 17], while structural alterations in the EC, including reduced volume have been reported in individuals following remission from MDD [18]. The EC integrates diverse sensory inputs, [19, 20] including olfactory information, and its neurons exhibit stimulus‐specific and state‐dependent firing patterns, suggesting a role in encoding experience‐dependent sensory representations [21, 22]. Notably, EC activity can modulate sensory throughput to limbic structures: stimulation of the EC suppresses olfactory input from the olfactory bulb to the BLA, and to a lesser extent the piriform cortex [23]. Given that the BLA encodes the motivational significance of sensory cues during associative learning [24], and that EC→BLA synapses exhibit activity‐dependent plasticity [25], this pathway is well positioned to shape the emotional valence of sensory experiences. Consistent with this, the EC contributes to multimodal sensory integration [26, 27], and the BLA represents a major downstream target of EC efferents [13, 28]. Importantly, EC→BLA projections are recruited during associative processing of aversive sensory cues and are required for encoding emotionally salient associations [29]. Furthermore, EC neurons projecting to the BLA are necessary for the retrieval of aversive and drug‐associated memories, underscoring a functional role for this circuit in encoding negative motivational states. Given that persistent negative memory bias is a core feature of depression, these findings implicate the EC→BLA circuit as a potential substrate for maladaptive emotional memory processes in depressive disorders [30].

A prominent feature of EC→BLA connectivity is the substantial contribution of neurons expressing the neuropeptide cholecystokinin (CCK) [31]. CCK signaling has been broadly implicated in neuropsychiatric disorders, including anxiety, post‐traumatic stress disorder (PTSD), and depression [32, 33, 34]. In humans, administration of the CCKB receptor agonist CCK‐4 induces acute panic attacks in both healthy individuals and patients with panic disorder [35], and elevated cerebrospinal fluid (CSF) levels of CCK have been reported in MDD [36]. At the circuit level, CCK‐expressing EC neurons project to the amygdala and are required for synaptic plasticity and associative fear memory formation [31]. Mechanistically, activation of EC→amygdala projections drives CCK release, which potentiates amygdala responses and facilitates experience‐dependent emotional learning. Moreover, CCK signaling within the EC→BLA pathway promotes stress susceptibility by enhancing synaptic plasticity in the BLA, thereby contributing to depressive‐like phenotypes. Pharmacological blockade of CCKB receptors in the BLA suppresses this plasticity and produces antidepressant‐like effects, highlighting CCKBR as a potential therapeutic target [37]. Despite these advances, the upstream molecular mechanisms that govern CCK release within the EC→BLA circuit, particularly under conditions of stress and depression, remain poorly defined.

An earlier study suggest that activation of metabotropic glutamate receptor 5 (mGluR5) inhibits CCK release in the hippocampus [38]. mGluR5 is a promising molecular candidate implicated in mood regulation. Reduced mGluR5 expression has been reported in postmortem tissue from individuals with MDD [39], as well as in animal models of chronic stress [40, 41]. Genetic deletion or pharmacological blockade of mGluR5 has been shown to enhance fear generalization and precipitate depressive‐like behaviors [42, 43, 44]. Despite these associations, the mechanistic link between mGluR5 signaling and CCK‐mediated neuromodulation in affective circuits remains unclear.

In this study, we employed a multidisciplinary approach, including anatomical tracing, in vitro electrophysiology, fiber photometry, optogenetics, pharmacology, and behavioral analyses, to investigate the role of mGluR5 in regulating depressive‐like behavior through CCK signaling in the EC^CCK^→BLA circuit. Our findings uncover a novel pathway by which mGluR5 modulates affective behavior via synaptic control of neuropeptide release, offering new insights into the cellular and circuit mechanisms underlying depression.

## 2 Results

### 2.1 EC^CCK^→BLA Circuit Encodes Negative Emotional Valence

**FIGURE 1:**
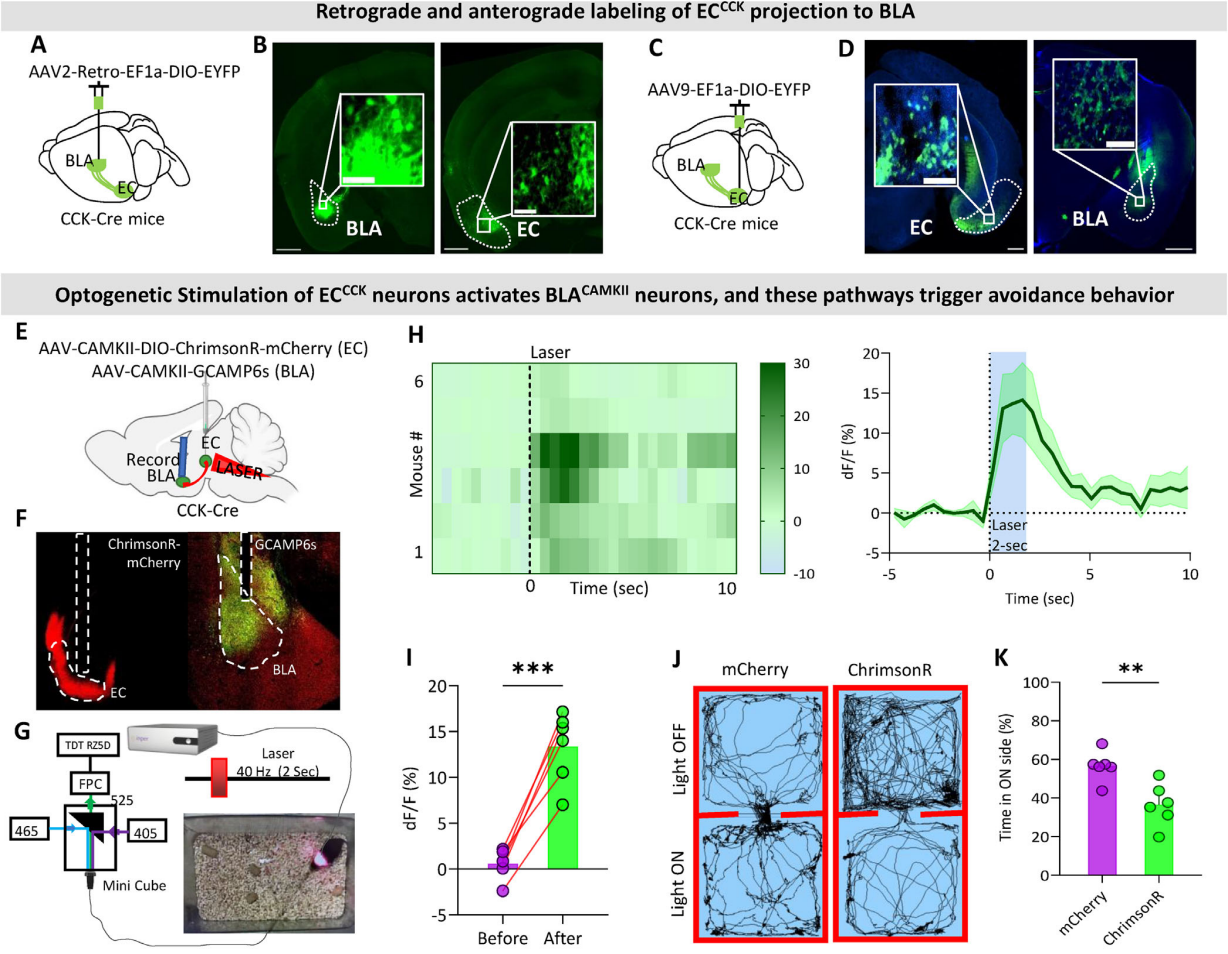
EC^CCK^ neurons project to BLA and encodes negative valence (**A)** Schematic representation of experiment design for retrograde labeling. (**B)** Left: injection site (BLA). Right: retrogradely labeled cells in the EC. **(C)** Experimental design for anterograde labeling. (**D)** Left: injection site (EC). Right: anterograde fibers in the BLA. **(E)** Experimental design. (**F)** Virus expression into EC ChrimsonR‐mCherry and BLA CAMKII‐GCAMP6s. (**G)** Setup for fiber photometry recording. (**H)** Left: Heat map; Right: Average calcium traces of BLA^Glu^ neurons before and during laser activation of EC^CCK^ neurons. (**I)** Average and individual responses of BLA^Glu^ neurons before and after laser stimulation of EC^CCK^ neurons. (**J)** Trajectory map for mCherry and ChrimsonR during real‐time place avoidance task. (**K)** Time spent on the laser on the side (%) among mCherry and ChrimsonR groups. All data are shown as mean ± s.e.m. *p < 0.05; **p < 0.01; ***p < 0.001; ****p < 0.0001 (see Table S1 for detailed statistics).

Some studies have identified the EC as a major input to the BLA, with EC neurons enriched in CCK expression [10, 13, 31]. To anatomically confirm the projection from EC^CCK^ neurons to the BLA, we performed both retrograde and anterograde viral tracing. Retro‐AAV2‐EF1a‐DIO‐EYFP was injected into the BLA and AAV9‐EF1a‐DIO‐EYFP into the EC of CCK‐Cre mice. Five weeks post‐injection, retrogradely labeled EC neurons and anterogradely labeled fibers in the BLA were observed, confirming a monosynaptic EC^CCK^→BLA projection (Figure 1A–D).

Building upon our prior finding that aversive stimuli activate BLA^Glu^ neurons and promote depressive‐like behavior [10], we hypothesized that EC^CCK^ neurons may modulate BLA^Glu^ activity and transmit aversive information. To test this, we co‐injected AAV‐CaMKII‐GCaMP6s into the BLA and AAV‐DIO‐ChrimsonR‐mCherry into the EC of CCK‐Cre mice. Optic fibers were implanted above both regions to enable selective activation of EC^CCK^ neurons while recording calcium activity from BLA^Glu^ neurons (Figure 1E–G). Optogenetic stimulation of EC^CCK^ neurons significantly increased calcium transients in BLA^Glu^ neurons, confirming rapid excitation (Figure 1H–I; Laser (Before versus After) 0.98 ± 0.32 vs. 13.71 ± 3.21). Given that BLA^Glu^ activation is sufficient to induce real‐time place aversion (RTPA) [10], we next tested whether EC^CCK^→BLA stimulation elicits similar behavioral responses. Indeed, optogenetic activation of EC^CCK^ terminals in the BLA significantly reduced time spent in a light‐paired chamber compared to controls (Figure 1J, K; mCherry 56.51 ± 3.16 vs. ChrimsonR 36.60 ± 4.46).

**FIGURE 2:**
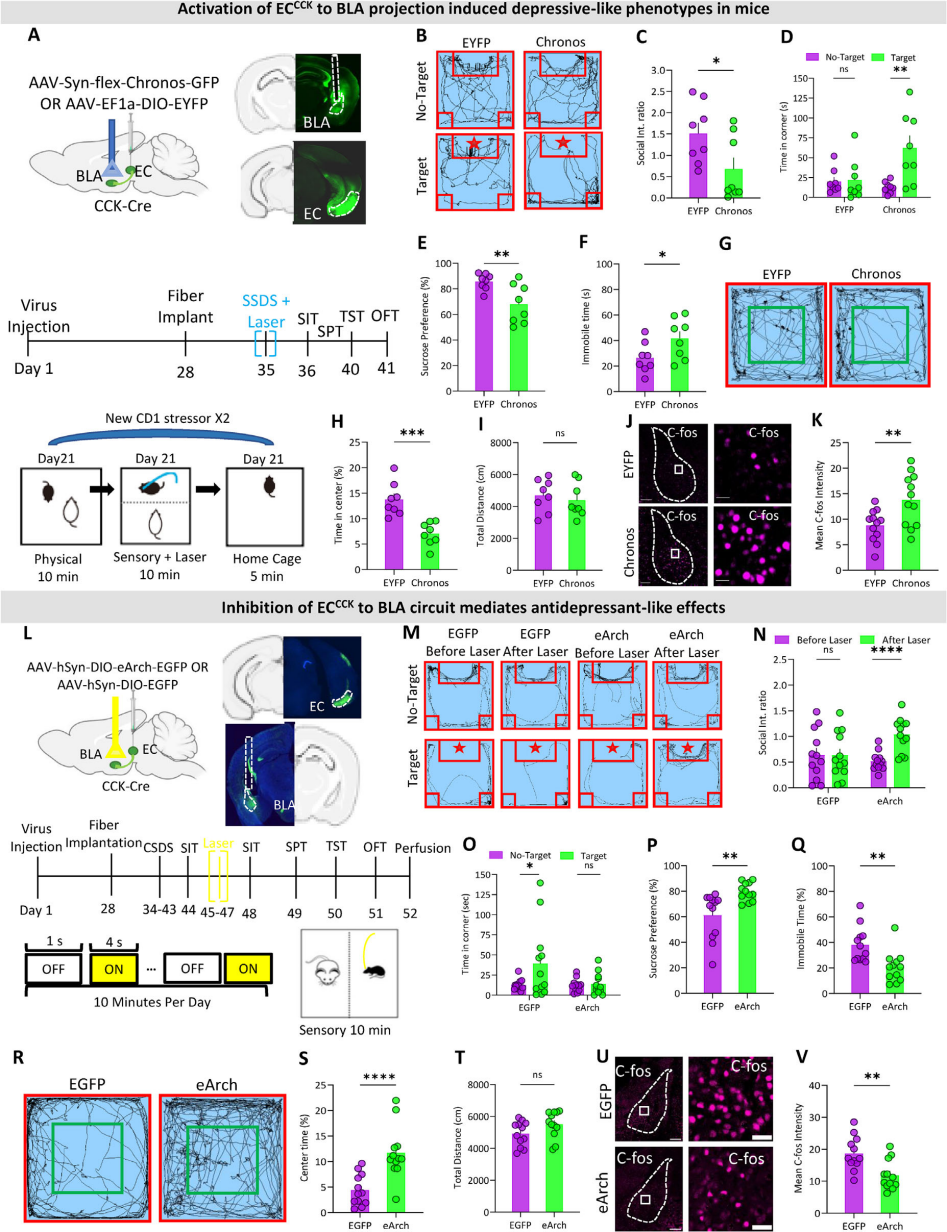
EC^CCK^‐BLA circuit mediates depressive‐Like behaviors (**A)** Top: Schematical illustration of the experimental design and virus expression in the EC (injection site) and BLA (terminals); Bottom: Experimental protocol for SSDS. (**B)** Showing the trajectory map of EYFP vs. Chronos in the presence of Target or No‐target. (**C)** Average social interaction ratio of EYFP vs. Chronos group. (**D)** Time in a corner (sec) in the presence of Target and No‐Target among EYFP and Chronos groups. (**E)** % Sucrose preference among EYFP vs. Chronos group. **(F)** Immobile duration during the tail suspension test between EYFP vs. Chronos group. (**G)** Trajectory map among EYFP vs. Chronos during OFT. (**H)** Time spent in center (%) in the OFT**. (I)** Total distance traveled during OFT between EYFP vs. Chronos. (**J)** Representative image of c‐fos expression; Left: enlarged 200um; right: zoomed 50um. (**K)** Average c‐fos intensity among EYFP vs. Chronos group. (**L)** Top: Schematical illustration of the experimental design and virus expression in the EC (injection site) and BLA (terminals); Bottom: Experimental protocol. (**M)** Showing the trajectory map of EGFP vs. eArch before and after laser activation in the presence of Target or No‐target. (**N)** Average social interaction ratio of EGFP vs. eArch group. (**O)** Time in a corner (sec) in the presence of Target and No‐Target among EGFP and eArch group**. (P)** % Sucrose preference between EGFP vs. eArch group. **(Q)** Total immobile time in TST between EGFP and eArch. (**R)** Trajectory map among EGFP and eArch during OFT. (**S)** Time spent in center (%) in the OFT**. (T)** Total distance traveled during OFT between EGFP and eArch. (**U)** Representative image of c‐fos expression; Left: enlarged 200um; right: zoomed 50um. (**V)** Average c‐fos intensity among EGFP and eArch group. All data are shown as mean ± s.e.m. *p < 0.05; **p < 0.01; ***p < 0.001; ****p < 0.0001 (see Table S1 for detailed statistics).

To determine whether this circuit contributes to depressive‐like behavior, we utilized a subthreshold social defeat (SSDS) paradigm, which alone is insufficient to induce robust depression‐like phenotypes [45]. CCK‐Cre mice received EC injections of AAV‐syn‐FLEX‐Chronos‐GFP or control AAV‐DIO‐EYFP, followed by BLA fiber implantation. After recovery, mice were subjected to SSDS. During each defeat session, EC^CCK^ terminals in the BLA were activated with 40 Hz laser pulses during a 10‐minute sensory exposure to CD‐1 aggressors (Figure 2A). Mice receiving EC^CCK^→BLA stimulation displayed increased susceptibility to stress, evidenced by reduced social interaction (Figure 2B, C; EYFP 1.51 ± 0.25 vs. Chronos 0.68 ± 0.27), decreased sucrose preference (Figure 2E; EYFP 85.28 ± 2.20 vs. Chronos 68.07 ± 5.44), increased immobility in the tail suspension test (TST) (Figure 2F; EYFP 26.49 ± 4.18 vs. Chronos 41.64 ± 5.59), and reduced center time in the open field test (OFT) (Figure 2G, H; EYFP 13.75 ± 1.15 vs. Chronos 7.08 ± 0.80), with no changes in total distance traveled (Figure 2I; EYFP 4698 ± 365.1 vs. Chronos 4391 ± 378.9). Furthermore, Chronos‐stimulated mice spent more time in the corners when a social target was present (Figure 2D; EYFP 22.01 ± 9.23 vs. Chronos 62.27 ± 15.48) and exhibited increased c‐Fos expression in the BLA (Figure 2J, K; EYFP 8.75 ± 0.89 vs. Chronos 13.77 ± 1.46).

To examine whether silencing the EC^CCK^→BLA pathway could alleviate depressive‐like behavior, we expressed the inhibitory opsin eArch in EC^CCK^ neurons (Figure 2L). Optogenetic inhibition of EC^CCK^ terminals in the BLA significantly improved social interaction in the CSDS model (Figure 2M, N; EGFP before laser 0.63 ± 0.14 vs. EGFP after laser 0.63 ± 0.13; eArch before laser 0.52 ± 0.05 vs. eArch after laser 1.04 ± 0.10), enhanced sucrose preference (Figure 2P; EGFP 61.16 ± 5.21 vs. eArch 79.58 ± 2.05), reduced TST immobility (Figure 2Q; EGFP 38.08 ± 4.03 vs. eArch 20.64 ± 3.49), and increased center time in the OFT (Figure 2R, S; EGFP 4.41 ± 0.88 vs. eArch 11.69 ± 1.48), without affecting locomotor activity (Figure 2T; EGFP 4919 ± 590.7 vs. eArch 5509 ± 328.6). Mice in the eArch group also spent less time in the corners in the presence of a target (Figure 2O; EGFP 39.36 ± 13.29 vs. eArch 13.7 ± 3.87), and showed reduced BLA c‐Fos expression (Figure 2U, V; EGFP 18.68 ± 1.51 vs. eArch 11.87 ± 1.33). Together, these gain and loss of function experiments demonstrate that the EC^CCK^→BLA circuit conveys aversive emotional signals and is sufficient to promote or suppress depressive‐like states, positioning EC^CCK^ neurons as a critical upstream driver of stress vulnerability via modulation of BLA activity.

### 2.2 mGluR5 is Enriched Postsynaptically in the EC^CCK^→BLA Circuit

**FIGURE 3:**
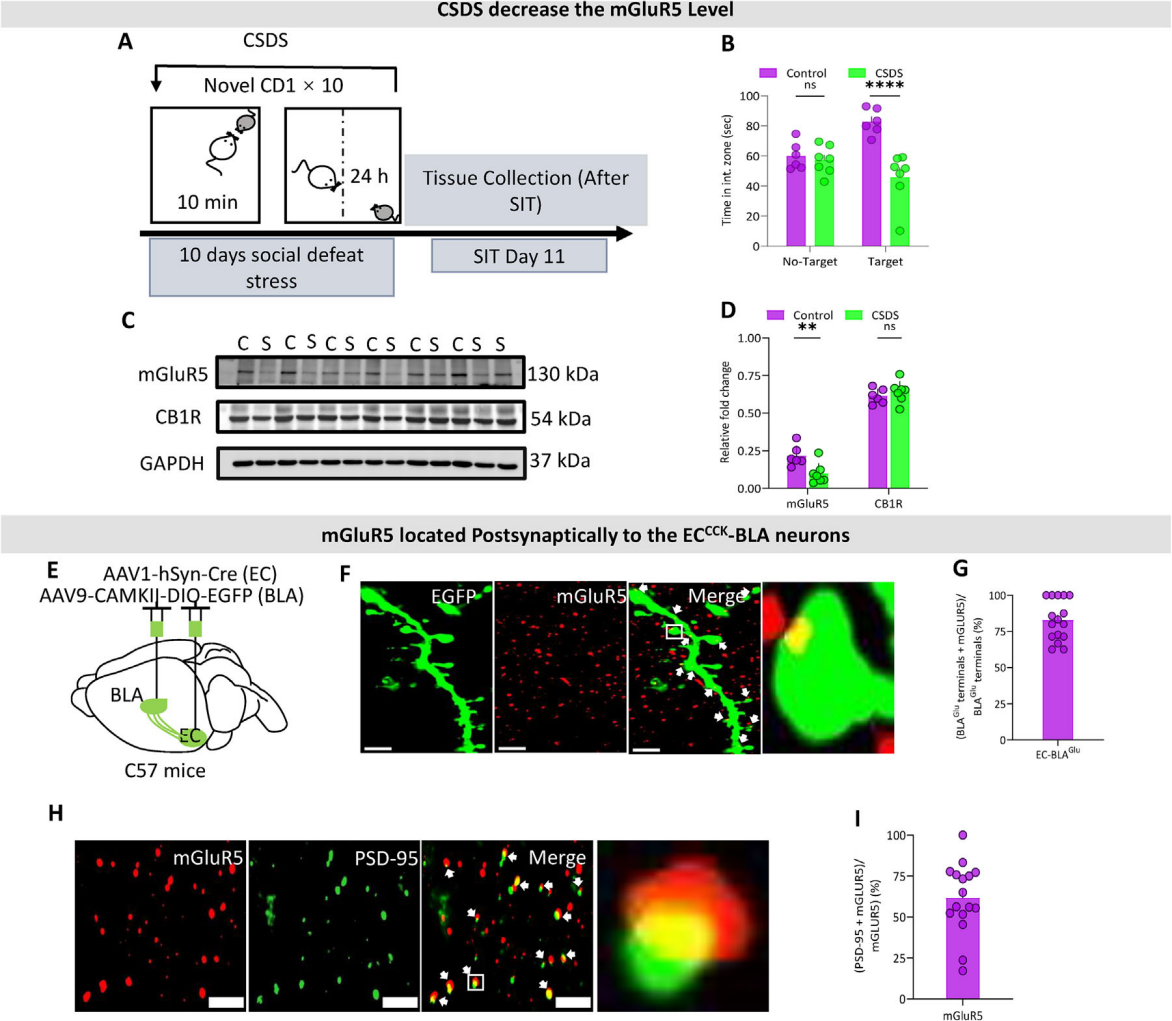
MGluR5 is located postsynaptically in the EC^CCK^‐BLA pathways. (**A)** Experimental design for chronic social defeat (CSDS). (**B)** Time spent in the interaction zone in the presence of target and No‐target among control and CSDS groups. (**C)** Western Blot image showing the decrease in mGluR5 level and no change in CB1R level in the CSDS group. (**D)** Showing the relative fold change of mGluR5 and CB1R among control and CSDS groups. (**E)** Schematic design for virus injection. (**F)** Image showing the colocalization of mGLUR5 with BLA^Glu^ neurons (terminals) which receiving input from EC. Left: BLA^Glu^ terminlas (Green), mGluR5 (red), Yellow (merge), Scale bar 10 um; Right: zoomed synapse with mGluR5 colocalization. (**G)** Average percentage of colocalized synapse with mGLUR5. (**H)** Image showing the colocalization of mGLUR5 with PSD‐95 (postsynaptic marker). Left: PSD‐95 (Green), mGluR5 (red), Yellow (merge), Scale bar 10 um; Right: zoomed image with mGluR5 and PSD‐95 colocalization. (**I)** Average percentage of colocalized PSD‐95 with mGLUR5. All data are shown as mean ± s.e.m. *p < 0.05; **p < 0.01; ***p < 0.001; ****p < 0.0001 (see Table S1 for detailed statistics). Also, See Figure S1.

Given the established role of mGluR5 in mood regulation [43, 44], its involvement in retrograde endocannabinoid (eCB) signaling through presynaptic cannabinoid receptor 1 (CB1R) [46, 47, 48], and the known expression of CB1R on CCK‐positive terminals [34, 49]. Moreover, our unpublished data showed that CB1R are located on presynaptic terminals of EC^CCK^ terminals in the BLA. Therefore, we sought to examine whether CSDS alters mGluR5 or CB1R levels in the BLA. Behaviorally, CSDS significantly reduced time spent in the interaction zone with a social target (Figure 3A, B; Control 82.83 ± 3.48 vs. CSDS 45.84 ± 6.42). Correspondingly, western blot analysis revealed a marked reduction in mGluR5 protein expression in the BLA (Figure 3C, D; Control 0.22 ± 0.03 vs. CSDS 0.098 ± 0.03), while CB1R levels remained unchanged (Figure 3C, D; Control 0.61 ± 0.02 vs. CSDS 0.64 ± 0.03).

To determine whether mGluR5 is expressed within the EC^CCK^→BLA pathway, we injected AAV1‐hSyn‐Cre into the EC and, two weeks later, AAV9‐CaMKII‐DIO‐EGFP into the BLA (Figure 3E). Four weeks post‐injection, expansion microscopy confirmed that EC projections target glutamatergic neurons in the BLA, as indicated by CaMKII labeling. Notably, these BLA^Glu^ neurons expressed high levels of mGluR5 on both somata and dendritic processes receiving EC input (Figure 3F, G; 82.75 ± 3.52, and Figure S1A, B), and mGluR5 was colocalized with the postsynaptic density marker PSD‐95 (Figure 3H, I; 61.60 ± 5.37), consistent with a postsynaptic localization of mGluR5 [50, 51]. To assess potential presynaptic expression of mGluR5, we injected AAV‐EF1a‐DIO‐mCherry into the EC of CCK‐Cre mice and performed expansion microscopy six weeks later (Figure S1C). EC^CCK^ terminals in the BLA expressed very few mGluR5 (Figure S1D, E; 16.12 ± 2.06), and this expression was significantly lower than that observed postsynaptically on BLA^Glu^ neurons (Figure S1F; BLA^Glu^ 82.75 ± 3.52 vs. EC^CCK^‐BLA 16.12 ± 2.06), suggesting a predominant postsynaptic localization.

### 2.3 mGluR5 Modulates Depressive‐Like Phenotypes Through CCK Signaling

**FIGURE 4:**
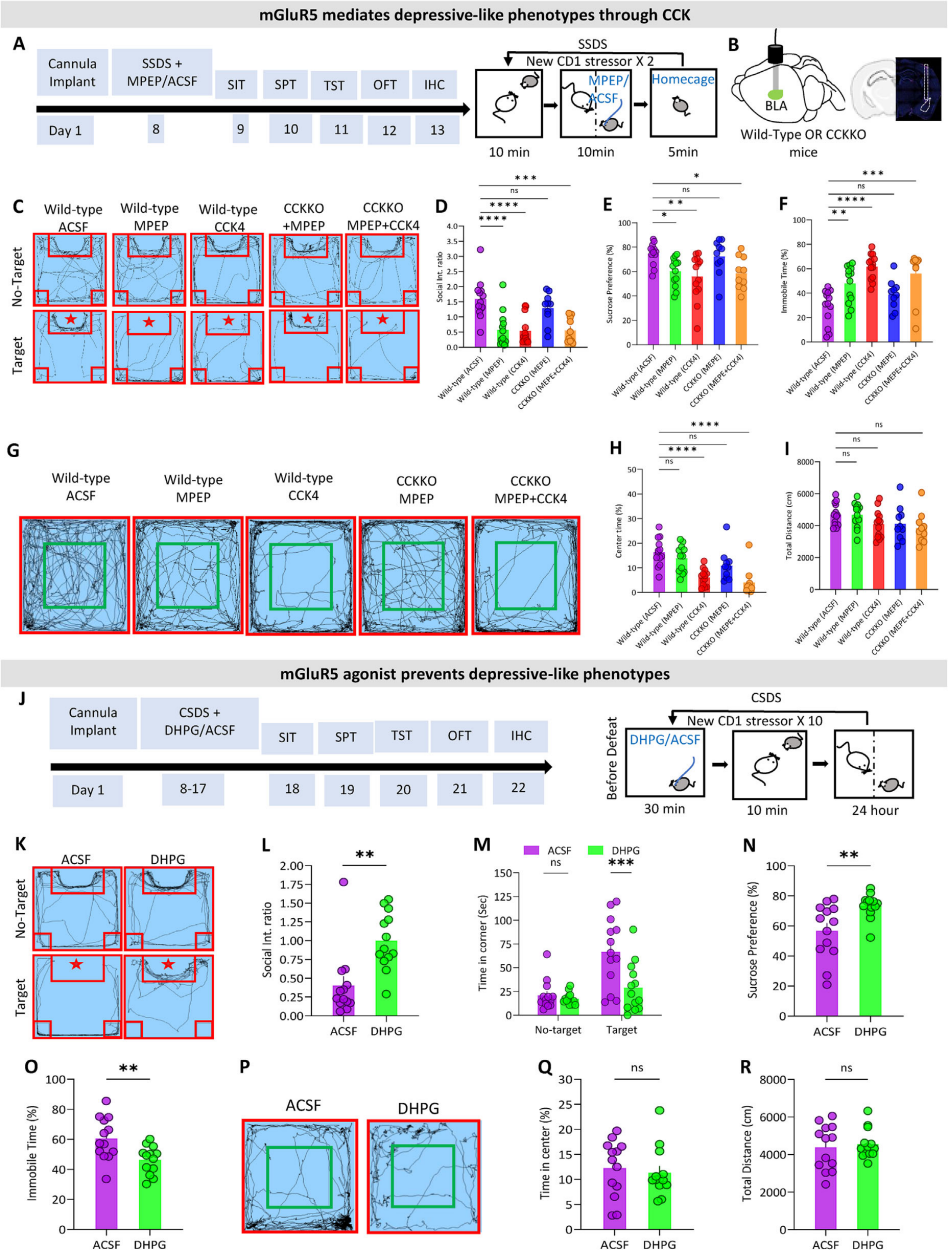
mGluR5 modulates depressive‐like phenotypes through CCK. (A) Experimental design and setup. (**B)** Representative image for cannula location. (**C)** Showing the trajectory map of Wildtype ACSF, Wildtype MPEP, Wildtype CCK4, CCKKO (MPEP), and CCKKO (MPEP+CCK4) in the presence of Target or No‐target during SIT. (**D)** Average social interaction ratio of Wildtype ACSF, Wildtype MPEP, Wildtype CCK4, CCKKO (MPEP), and CCKKO (MPEP+CCK4) group. (**E)** % Sucrose preference among Wildtype ACSF, Wildtype MPEP, Wildtype CCK4, CCKKO (MPEP), and CCKKO (MPEP+CCK4) group. **(F)** Immobile duration during the tail suspension test between Wildtype ACSF, Wildtype MPEP, Wildtype CCK4, CCKKO (MPEP), and CCKKO (MPEP+CCK4) group. (**G)** Trajectory map among Wildtype ACSF, Wildtype MPEP, Wildtype CCK4, CCKKO (MPEP), and CCKKO (MPEP+CCK4) during OFT. (**H)** Time spent in center (%) in the OFT. (**I)** Total distance traveled during OFT. (**J)** Experimental design and setup. (**K)** Showing the trajectory map of ACSF vs. DHPG in the presence of Target or No‐target. (**L)** Average social interaction ratio of ACSF vs. DHPG group. (**M)** Time spent in a corner among ACSF vs. DHPG in the presence of Target or No‐target during SIT. (**N)** % Sucrose preference among ACSF vs. DHPG group. **(O)**. Immobile duration during the tail suspension test between ACSF vs. DHPG group. **(P)**. Trajectory map among ACSF vs. DHPG during OFT. (**Q)** Time spent in center (%) in the OFT. (**R)** Total distance traveled during OFT. All data are shown as mean ± s.e.m. *p < 0.05; **p < 0.01; ***p < 0.001; ****p < 0.0001 (see Table S1 for detailed statistics). Also, see Figure S2.

To determine whether mGluR5 contributes to stress susceptibility through a CCK‐dependent mechanism, we selectively blocked mGluR5 in the BLA using local infusion of the antagonist MPEP in both wild‐type and CCK knockout (CCKKO) mice during SSDS (Figure 4A, B). Strikingly, CCK was required for the expression of MPEP‐induced depressive‐like behaviors, suggesting that mGluR5 modulates affective states via CCK signaling in the BLA. In the Social Interaction Test (SIT), MPEP administration in wild‐type mice significantly reduced the social interaction ratio compared to vehicle controls, while this effect was abolished in CCKKO mice. Reintroduction of CCK (via CCK‐4) in CCKKO animals restored the behavioral phenotype (Figure 4C, D; Wild‐type ACSF: 1.59 ± 0.18 vs. Wild‐type MPEP: 0.58 ± 0.16; Wild‐type CCK4: 0.54 ± 0.13; CCKKO MPEP: 1.29 ± 0.16; CCKKO MPEP+CCK4: 0.56 ± 0.13). Similarly, in the Sucrose Preference Test (SPT), MPEP and CCK4 significantly reduced sucrose consumption in wild‐type mice, whereas CCKKO mice maintained higher preference following MPEP treatment. This antidepressant‐like profile was reversed by CCK4 administration in CCKKO mice (Figure 4E; Wild‐type ACSF: 74.61 ± 2.46 vs. Wild‐type MPEP: 60.18 ± 3.24; Wild‐type CCK4: 55.79 ± 5.17; CCKKO MPEP: 72.30 ± 4.64; CCKKO MPEP+CCK4: 58.51 ± 4.18). Consistent results were observed in the TST, where immobility time increased following MPEP or CCK4 in wild‐type mice, but not in CCKKO mice treated with MPEP alone. The depressive‐like phenotype was reinstated in CCKKO animals upon co‐administration of CCK4 (Figure 4F; Wild‐type ACSF: 29.01 ± 3.94 vs. Wild‐type MPEP: 47.92 ± 4.23; Wild‐type CCK4: 61.73 ± 2.87; CCKKO MPEP: 38.85 ± 3.82; CCKKO MPEP+CCK4: 55.89 ± 6.49). In the presence of a social target, MPEP and CCK4 increased time spent in the corners in wild‐type mice, consistent with social avoidance, whereas CCKKO mice displayed reduced corner time that was rescued by CCK4 (Figure S2A; Wild‐type ACSF with Target: 16.47 ± 3.06 vs. Wild‐type MPEP with Target: 58.43 ± 8.21; Wild‐type CCK4 with Target: 72.38 ± 15.99; CCKKO MPEP with Target: 39.54 ± 12.96; CCKKO MPEP+CCK4 with Target: 83.58 ± 15.75). Anxiety‐like behavior in the OFT was also modulated by CCK signaling. MPEP and CCK4 reduced time spent in the center in wild‐type mice, while CCKKO mice maintained higher center time after MPEP treatment. CCK4 reinstated anxiety‐like behavior in these animals (Figure 4G, H; Wild‐type ACSF: 16.34 ± 1.55 vs. Wild‐type MPEP: 13.82 ± 1.5; Wild‐type CCK4: 6.11 ± 0.93; CCKKO MPEP: 10.90 ± 2.03; CCKKO MPEP+CCK4: 4.08 ± 1.79). Total distance traveled was comparable across groups, indicating that the observed effects were not due to alterations in locomotion (Figure 4I; Wild‐type ACSF: 4698 ± 194.2 vs. Wild‐type MPEP: 4685 ± 214.6; Wild‐type CCK4: 4095 ± 240.5; CCKKO MPEP: 4118 ± 346.2; CCKKO MPEP+CCK4: 3852 ± 307.7). Finally, immunohistochemical analysis revealed that c‐Fos expression in the BLA, a marker of neuronal activation, was significantly increased by MPEP and CCK4 in wild‐type mice, but not in CCKKO animals treated with MPEP. This activity was restored upon CCK4 co‐administration (Figure S2B, C; Wild‐type ACSF: 7.81 ± 0.52 vs. Wild‐type MPEP: 12.90 ± 0.63; Wild‐type CCK4: 12.43 ± 1.42; CCKKO MPEP: 9.66 ± 0.85; CCKKO MPEP+CCK4: 15.05 ± 1.12).

### 2.4 mGluR5 Agonist Prevent Depression‐Like Behaviors Induced by Chronic Social Defeat Stress

To examine whether mGluR5 activation confers resilience to stress‐induced depressive‐like behaviors, we administered the mGluR5 agonist DHPG in a CSDS paradigm. Mice received daily intra‐BLA infusions of DHPG 30 min prior to each defeat episode for 10 consecutive days (Figure 4J). DHPG treatment significantly prevent CSDS‐induced behavioral deficits. Compared to ACSF controls, DHPG‐treated mice exhibited a higher social interaction ratio in the SIT (Figure 4K, L; ACSF: 0.40 ± 0.12 vs. DHPG: 0.99 ± 0.10), greater sucrose preference in the SPT (Figure 4N; ACSF: 56.71 ± 5.13 vs. DHPG: 73.41 ± 2.24), and reduced immobility time in the TST (Figure 6O; ACSF: 60.57 ± 3.93 vs. DHPG: 46.40 ± 2.57). Additionally, DHPG reduced corner time during the SIT in the presence of a social target, reflecting decreased social avoidance (Figure 4M; ACSF: 66.64 ± 10.14 vs. DHPG: 29.24 ± 7.21). No significant differences were observed in center time (Figure 4P, Q; ACSF: 12.24 ± 1.58 vs. DHPG: 11.34 ± 1.36; p<0.6) or total distance traveled in the OFT (Figure 4R; ACSF: 4381 ± 334.2 vs. DHPG: 4557 ± 216.4), indicating that the effects of DHPG were not attributable to changes in general locomotor activity or anxiety‐like behavior. Furthermore, DHPG‐treated mice showed significantly reduced c‐Fos expression in the BLA relative to ACSF controls, suggesting decreased neuronal activation (Figure S2D, E; ACSF: 14.45 ± 1.02 vs. DHPG: 9.18 ± 0.56). These findings demonstrate that mGluR5 activation dampens stress‐induced behavioral and neural activities in the BLA, implicating mGluR5 signaling may be a protective modulator against the development of depressive‐like phenotypes.

### 2.5 mGluR5 Regulates the Synaptic Plasticity in the BLA Via CCK

**FIGURE 5:**
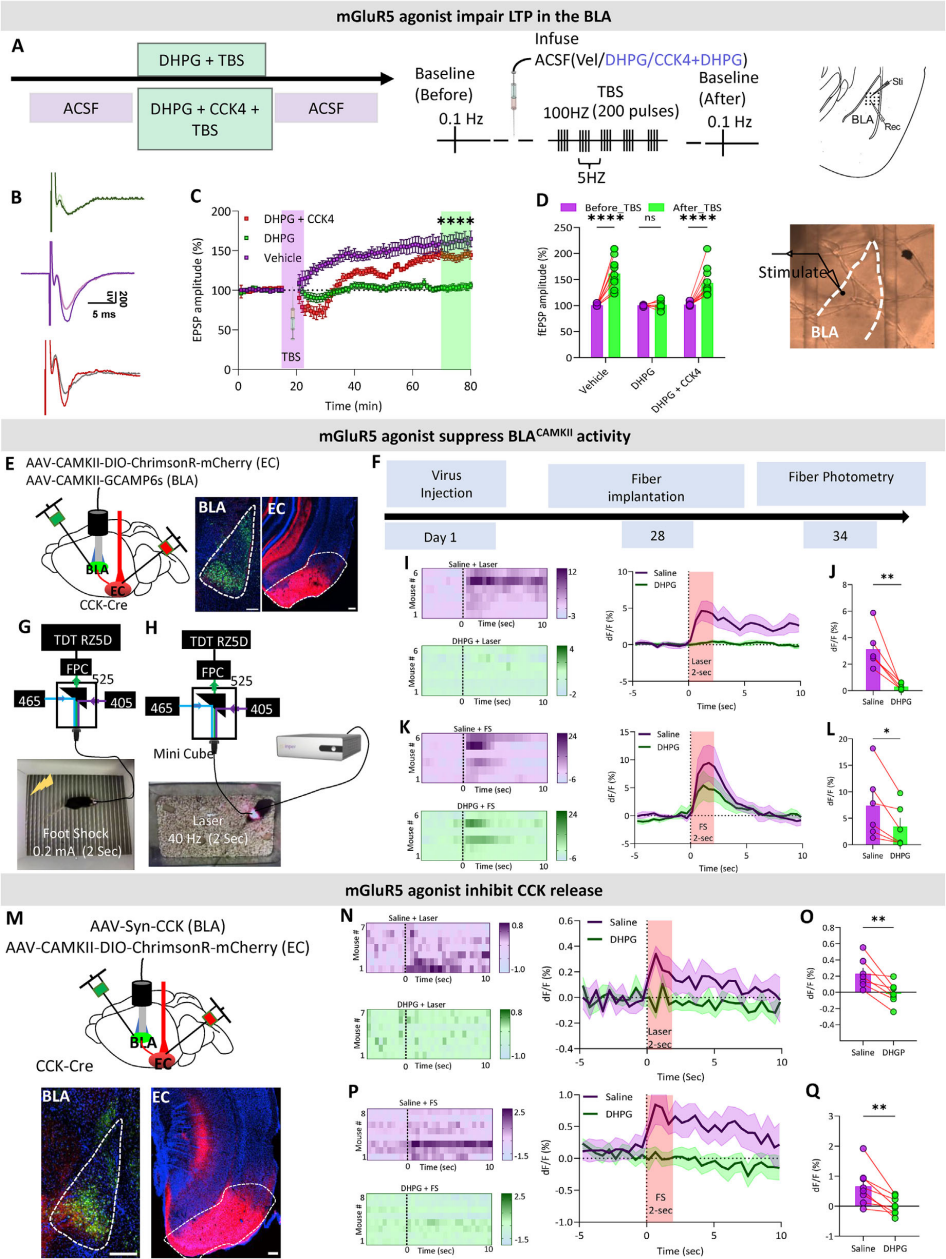
MGluR5 agonist inhibit CCK release and impair LTP in the BLA. (**A)** Top Left: Schematic representation of the experimental design. Top right: schematic diagram of baseline and TBS. Bottom right: light microscopy photograph showing the location of a 4*4 microelectrode array placed on the BLA region. (**B)** Representative of single field excitatory postsynaptic potential (fEPSP) before and after TBS in vehicle, DHPG, and DHPG + CCK4. (**C)** % fEPSPs amplitude traces before and after TBS in a vehicle (purple), DHPG (Green), DHPG + CCK4 (Red). (**D)** Average change in fEPSPs (%) before and after TBS in vehicle, DHPG, and DHPG + CCK4. (**E)** Left: Experimental design. Right: representative image for virus expression, EC is injected with ChrimsonR (Red), BLA injected with GCAMP6s (Green), scale bar set to 200 um. (**F)** Experimental schedule. (**G)** Experimental setup for fiber photometry recording during laser activation of EC^CCK^ neurons. (**H)** Showing the setup for fiber photometry recording during footshock (FS) stimulation. (**I)** Left: Heat map; Right: Average calcium traces before and during laser activation of EC^CCK^ neurons. (**J)** Individual responses among saline and DHPG groups during laser activation. (**K)** Left: Heat map; Right: Average calcium traces before and during footshock (FS). (**L)** Individual responses among saline and DHPG groups during footshock (FS). (**M)** Top: Experimental design. Bottom: representative image for virus expression, EC is injected with ChrimsonR (Red), BLA injected with GCAMP6s (Green), scale bar set to 200 um. (**N)** Left: Heat map; Right: Average CCK traces before and during laser activation of EC^CCK^ neurons. (**O)** Individual CCK responses among saline and DHPG groups during laser activation. (**P)** Left: Heat map; Right: Average CCK traces before and during footshock (FS). (**Q)** Individual CCK responses among saline and DHPG group during footshock (FS). All data are shown as mean ± s.e.m. *p < 0.05; **p < 0.01; ***p < 0.001; ****p < 0.0001 (see Table S1 for detailed statistics). Also, See Figure S3.

Considering the postsynaptic localization of mGluR5 near EC^CCK^ terminals, the inhibitory effect of mGluR5 activation on CCK release [38], and the critical role of CCK in long term potentiation (LTP) induction [31, 52, 53, 54, 55], we hypothesized that mGluR5 may regulate synaptic plasticity in the BLA. To test this, we conducted ex vivo electrophysiological recordings in acute brain slices using microelectrode arrays, as described previously [37]. We compared theta‐burst stimulation (TBS)‐induced LTP in the BLA under three conditions: vehicle treatment, application of the mGluR5 agonist DHPG (100 µM), and co‐application of DHPG with the CCKB receptor agonist CCK‐4 (Figure 5A). DHPG significantly impaired TBS‐induced LTP compared to vehicle‐treated controls (Figure 5B—D; Vehicle: 161.35 ± 8.92 vs. DHPG: 101.66 ± 2.98). However, this impairment was rescued by co‐application of CCK‐4 (Figure 5B—D; DHPG: 101.66 ± 2.98 vs. DHPG + CCK4: 143.53 ± 8.24), suggesting that mGluR5‐mediated suppression of LTP involves inhibition of CCK release. Consistent with this mechanism, we also found that CCK knockout (CCK–^/–^) mice exhibited a marked deficit in TBS‐induced LTP in the BLA relative to wild‐type controls (Figure S3A–D; CCK‐/‐ 108.21 ± 2.27 vs. Control 170.06 ± 9.91 after TBS), further reinforcing the importance of CCK in synaptic potentiation.

### 2.6 mGluR5 Regulates CCK Release in the EC^CCK^→BLA Circuit

To further elucidate the mechanistic relationship between mGluR5 and CCK signaling, we investigated synaptic transmission in the EC^CCK^→BLA circuit by assessing the input‐output dynamics between optogenetic stimulation of EC^CCK^ neurons and calcium responses in BLA^Glu^ neurons following administration of saline or the mGluR5 agonist DHPG. We first injected AAV‐CaMKII‐GCaMP6s into the BLA and AAV‐CaMKII‐DIO‐ChrimsonR‐mCherry into the EC of CCK‐Cre mice. Four weeks later, an optical fiber was implanted over the BLA, and one week thereafter, fiber photometry recordings were conducted (Figure 5E–H). Thirty minutes prior to the recordings, mice were injected with either saline or DHPG. In saline‐treated animals, optogenetic activation of EC^CCK^ neurons reliably evoked calcium transients in BLA^Glu^ neurons. In contrast, DHPG‐treated mice showed markedly reduced responses (Figure 5I, J; Saline 3.13 ± 0.60 vs. DHPG 0.31 ± 0.10), indicating that mGluR5 activation suppresses excitatory drive from EC^CCK^ inputs to the BLA. Similarly, we observed that acute footshock stimulation robustly activated BLA^Glu^ neurons in the saline group, whereas this response was significantly blunted by DHPG administration (Figure 5K, L; Saline 7.34 ± 2.59 vs. DHPG 3.40 ± 1.63). Administration of the mGluR5 antagonist MPEP showed a trend toward enhancing footshock‐evoked BLA^Glu^ activity, though this did not reach statistical significance (Figure S3E, F; Saline 8.75 ± 3.50 vs. MPEP 11.87 ± 5.58).

To determine whether mGluR5 modulates CCK release in the EC^CCK^→BLA pathway, we co‐injected pAAV‐hSyn‐CCK2.3 into the BLA and AAV‐CaMKII‐DIO‐ChrimsonR‐mCherry into the EC of CCK‐Cre mice (Figure 5M). Optogenetic stimulation of EC^CCK^ neurons induced a robust increase in CCK sensor fluorescence in the BLA of saline‐treated mice, an effect that was significantly reduced in the presence of DHPG (Figure 5N, O; Saline 0.23 ± 0.07 vs. DHPG ‐0.02 ± 0.05). DHPG also suppressed footshock‐induced CCK release in the BLA (Figure 5P, Q; Saline 0.66 ± 0.21 vs. DHPG 0.02 ± 0.10). Analysis of averaged and individual traces confirmed a significant elevation in CCK release in the saline group after both footshock and laser stimulation compared to baseline, whereas DHPG‐treated animals failed to show any such increase (Figure S3G, H, Saline (Baseline vs footshock) 0.16 ± 0.14 vs 0.67 ± 0.21; DHPG (Baseline vs footshock) 0.06 ± 0.09 vs 0.013 ± 0.1). Moreover, DHPG treatment resulted in a diminished fluorescence signal relative to MPEP (Figure S3I–J; Saline (Baseline vs Laser) ‐0.03 ± 0.05 vs 0.23 ± 0.07; DHPG (Baseline vs Laser) 0.01 ± 0.04 vs ‐0.01 ± 0.05). These findings demonstrate that mGluR5 activation suppresses synaptic drive from EC^CCK^ neurons to the BLA and inhibits CCK release, indicating a critical role for mGluR5 in modulating this neuropeptidergic circuit.

### 2.7 mGluR5 Knockout in the EC^CCK^ to BLA Circuit Increases Susceptibility to Stress

**FIGURE 6:**
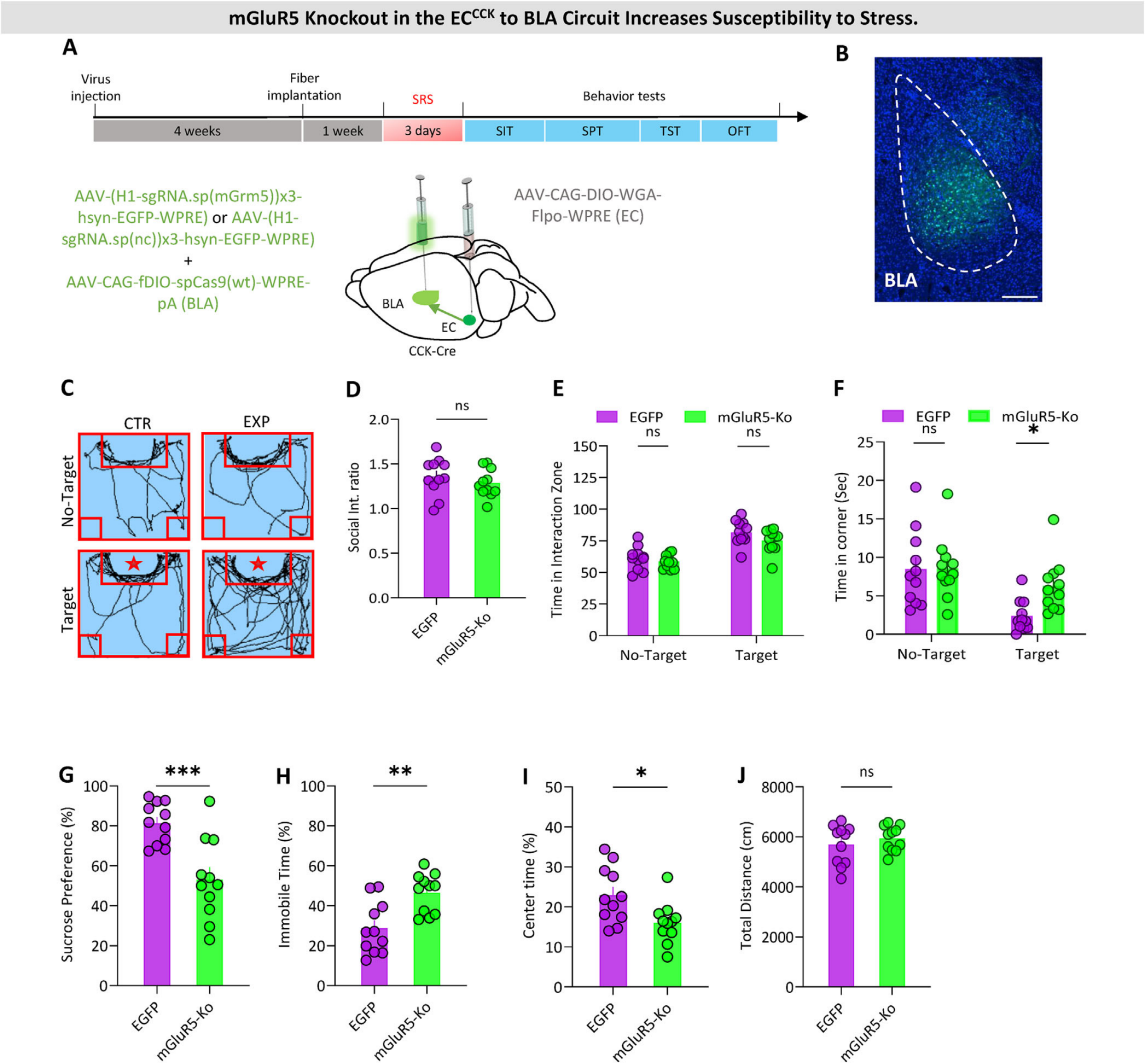
mGluR5 Knockout in the EC^CCK^ to BLA Circuit Increases Susceptibility to Stress. (A) Experimental design and setup. (**B)** Virus expression. (**C)** Showing the trajectory map of Control (EGFP) and Experimental group (mGluR5‐ko) in the presence of Target or No‐target during SIT. (**D)** Average social interaction ratio between EGFP and mGluR5‐Ko groups. (**E)** Time in interaction zone between EGFP and mGluR5‐Ko groups. **(F)** Time in corner between EGFP and mGluR5‐Ko groups. (**G)** Percentage of Sucrose preference between EGFP and mGluR5‐Ko groups during SPT. **(H)** Immobile time between EGFP and mGluR5‐Ko groups during TST. (**I)** Time spent in center (%) in the OFT. (**J)** Total distance traveled during OFT. All data are shown as mean ± s.e.m. *p < 0.05; **p < 0.01; ***p < 0.001; ****p < 0.0001 (see Table S1 for detailed statistics). Also, see Figure S4.

Next, we asked if EC to BLA circuit specific knockout of mGluR5 (mGluR5‐ko) increased the stress susceptibility. We adopted a candidate approach [37], to the circuit specifically the knockout of mGluR5. We selectively knocked out the mGluR5 in the EC‐BLA projection by injecting a anterogradely transported virus AAV‐CAG‐DIO‐WGA‐Flpo‐WPRE into the EC and virus encoding the mGluR5 guide RNA (AAV‐(H1‐sgRNA.sp(mGrm5))x3‐hsyn‐EGFP‐WPRE) together with virus encoding Cas9 (AAV‐CAG‐fDIO‐spCas9(wt)‐WPRE‐pA) into BLA. For control group (AAV‐(H1‐sgRNA.sp(nc))x3‐hsyn‐EGFP‐WPRE) was injected instead of (AAV‐(H1‐sgRNA.sp(mGrm5))x3‐hsyn‐EGFP‐WPRE) to assessed the effect of EC‐BLA mGluR5‐Ko on stress susceptibility (Figure 6A, B). We verified that our CRISPR‐Cas9 approach efficiently knockout the mGluR5 in EC‐BLA circuit (Figure S4; EGFP vs mGluR5‐ko). Indeed, EC‐BLA mGluR5‐ko increased stress susceptibility, which is indicated by increased time in the corner zone during social interaction assay (Figure 6C–F; EGFP vs mGluR5‐ko with a target, 19.4 ± 5.04 s vs. 52.1 ± 4.1 s), decreased sucrose preference (Figure 6G; EGFP vs mGluR5‐ko, 73.7 ± 2.02 vs. 83.3 ± 1.86), increased immobile time in TST (Figure 6H; EGFP vs mGluR5‐ko, 9.8 ± 1.62 vs. 15.7 ± 1.92), and decreased center time in OFT (Figure 6I, J; EGFP vs mGluR5‐ko) in mGluR5‐ko mice compared with control EGFP group mice.

### 2.8 CB1R Antagonist Prevent mGluR5 Agonist‐Mediated LTP Deficits

**FIGURE 7:**
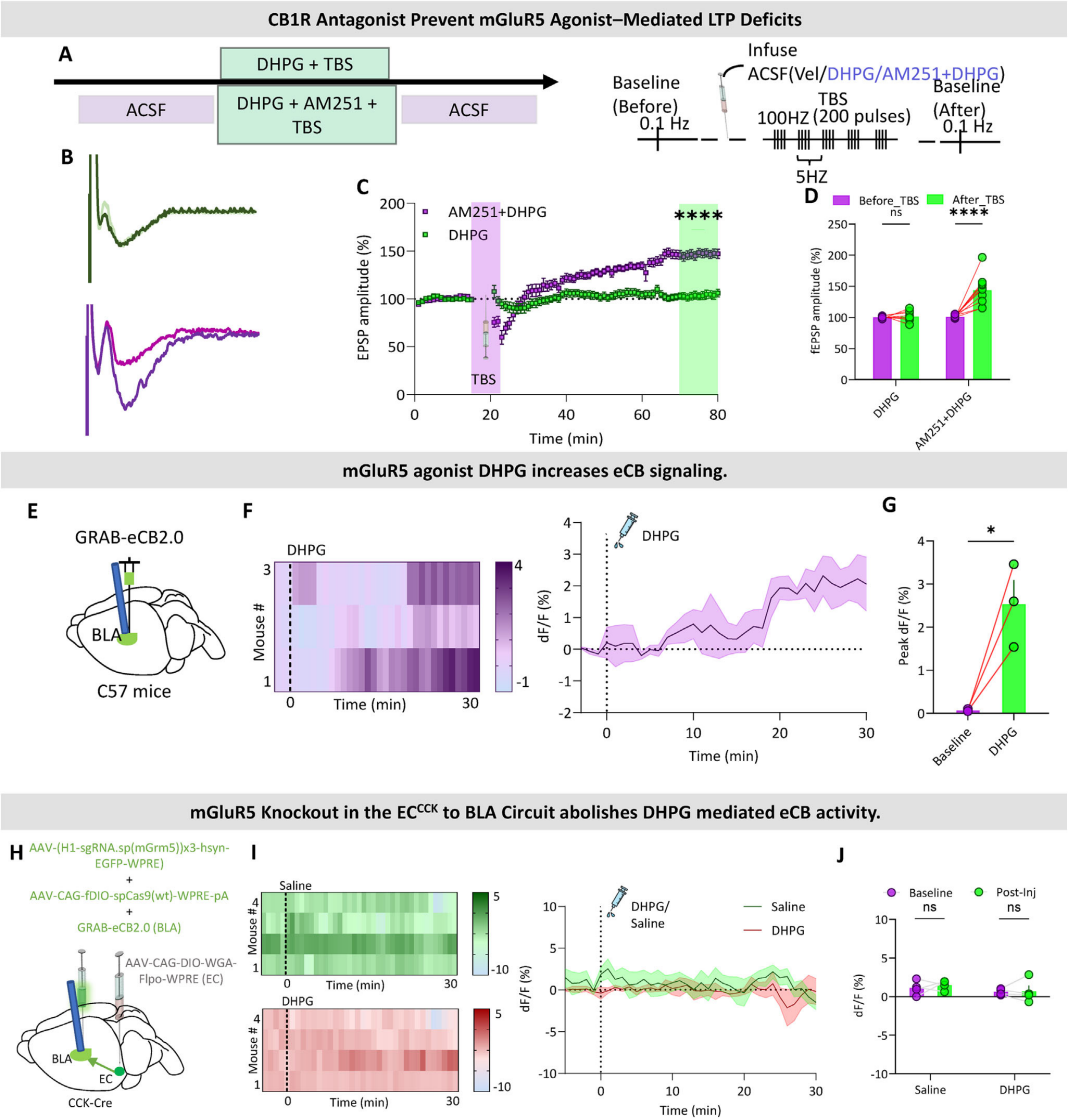
mGluR5 agonist activate eCB‐CB1R, and CB1R Antagonist Prevent mGluR5 Agonist–Mediated LTP Deficits. (**A**) Schematic representation of the experimental design. (**B)** Representative of single field excitatory postsynaptic potential (fEPSP) before and after TBS in DHPG and AM251+DHPG. (**C)** % fEPSPs amplitude traces before and after TBS in a DHPG (Green) and AM251+DHPG (Purple). (**D)** Average change in fEPSPs (%) before and after TBS in DHPG and AM251+DHPG. (**E)** Experimental design. **(F)** Left: Heatmap; Right: Average eCB traces before and after Injection of DHPG.(**G)** Average and individual change in eCB sensor during Baseline vs. last minute of recording. (**H**) Experimental design. (**I)** Left: Heatmap; Right: Average eCB traces before and after Injection of DHPG or saline in mGluR5‐ko groups. (**J)** Average and individual change in eCB sensor during Baseline vs. last minute of recording. All data are shown as mean ± s.e.m. *p < 0.05; **p < 0.01; ***p < 0.001; ****p < 0.0001 (see Table S1 for detailed statistics). Also, See Figure S5.

CB1Rs are well‐known components of CCK‐positive presynaptic terminals [34, 49, 56], and our data confirm their expression on EC^CCK^ terminals in the BLA (Figure S5A–C). Given that mGluR5 activation can initiate eCB release leading to CB1R‐mediated presynaptic inhibition [46, 47, 48], we ask whether mGluR5‐mediated effects are dependent on endocannabinoid signaling. Application of the CB1R antagonist AM251 prevented DHPG‐induced LTP impairment (Figure 7A–D; DHPG: 101.66 ± 2.98 vs. AM251+DHPG: 143.59 ± 7.56), implicating retrograde eCB signaling in this process. Supporting this, fluorescence‐based imaging with an endocannabinoid sensor revealed that DHPG application significantly increased CB1R activation (Figure 7E–G; Basleine ‐1.18 ± 0.80 vs DHPG 0.88 ± 1.17). Moreover, EC^CCK^→BLA mGluR5 knockout abolished the DHPG‐induced increase in eCB signaling (Figure 7H–J). Consistently, saline control injections did not produce any significant changes in eCB signaling (Figure 7H–J). Together, these findings indicate that mGluR5 activation could suppresses CCK release via retrograde eCB signaling at presynaptic CB1Rs, thereby regulating LTP induction in the BLA. This mGluR5–eCB–CCK signaling axis may serve as a critical molecular mechanism underlying synaptic plasticity and affective behavior in stress‐related disorders.

## 3 Discussion

Our findings reveal that activation of EC^CCK^ neurons enhances BLA excitability and drives the expression of depressive‐like behaviors, whereas inhibition of this circuit mitigates such phenotypes. These results align with previous reports implicating BLA hyperactivity and EC→BLA projections in the encoding of negative emotional states [31, 57, 58]. We further demonstrate that chronic stress leads to a downregulation of mGluR5 expression in the amygdala, consistent with prior studies [39, 40, 41]. Notably, mGluR5 is localized postsynaptically in close proximity to EC^CCK^ terminals within the BLA. Functionally, we show that mGluR5 regulates depressive‐like behaviors through a CCK‐dependent mechanism: pharmacological activation of mGluR5 suppresses CCK release and disrupts LTP in the BLA. Together, these findings identify the mGluR5–CCK signaling axis within the EC^CCK^→BLA circuit as a critical modulator of affective state, providing novel mechanistic insight and a potential target for therapeutic intervention in mood disorders.

### 3.1 Distinct Role of EC^CCK^→BLA Pathway in Depression

Despite the clinical [59, 60] and preclinical [57, 58] studies reported amygdala hyperactivity in depression, less attention has been given to understanding the mechanisms underlying amygdala hyperactivation in depression. In our previous work, we identified BLA^Glu^ hyperactivation as a marker of negative emotional states [10, 61], and we established that the BLA receives sensory inputs from multiple modalities [10, 11], with a robust projection from the EC that is enriched with CCK [10, 31, 62]. In this study, we demonstrate that the EC^CCK^ neurons project to the BLA, and their activation rapidly and strongly activates the BLA. Given that the processing of negative valence is known to regulate negative emotional states such as fear, anxiety, and depression, we observed that activation of the EC^CCK^→BLA circuit elicits extremely intense negative emotions compared to previously investigated pathways, such as LC→BLA [63], VTA→BLA [64], and IC→BLA [65]. Notably, RTPA tests revealed that EC^CCK^ → BLA activation resulted in the most significant aversion, suggesting that this circuit encodes potent negative valence. Inhibiting this pathway alleviated depression‐like behaviors, positioning the EC^CCK^ → BLA circuit as a distinctive therapeutic target for mood disorders.

### 3.2 Role of CCK in Depression

Previous studies have emphasized the crucial role of CCK in various forms of learning and memory, including fear [31], spatial [66], motor [67], and visual‐auditory associative memory formation [52, 62, 68]. While earlier research has highlighted the involvement of CCK and CCKB receptors in the regulation of anxiety and depression [69, 70, 71, 72, 73], most of these studies were limited to acute behavioral tests and did not explore the underlying mechanisms of CCK‐dependent modulation of behavior. Recently, we demonstrated that CSDS activates CCKBR in the BLA, and blocking CCKBR impairs LTP formation while exerting antidepressant‐like effects [37]. In our current study, we have discovered that the EC^CCK^→BLA pathways play a role in the modulation of depression through CCK, and CCK is essential for the development of depressive‐like phenotypes mediated by mGluR5 antagonism. While previous research has primarily focused on classical neurotransmitters and neuromodulators such as glutamate, GABA, serotonin, and dopamine in stress‐induced depressive disorders [11, 74, 75], it is worth noting that CCK receptors are expressed in regions implicated in depression, such as the ventral tegmental area (VTA), dorsal raphe nucleus (DRN), and nucleus accumbens (NAc) [76]. Hence, CCK may interact with these neurotransmitters or neuromodulators through its receptors, regulating the release of dopamine and serotonin in the brain and thereby influencing depressive disorders. In fact, a previous study demonstrated that CCK neurons in the VTA modulate dopamine release [77]. Furthermore, previous research has shown that neurons in the central amygdala (CeA) project to the VTA [78] and DRN [79]. In our current study, we found that CCK release in the BLA increases amygdala activity. Thus, an additional potential downstraem mechanism could involve CeA GABAergic projections to the DRN and VTA, inhibiting the release of serotonin and dopamine, respectively, and thereby triggering depression or related mood disorders. Further investigations are warranted to elucidate these mechanisms in detail. Additionally, exploring the role of CCK in other brain regions and neural circuits would be of great interest for a comprehensive understanding of its broader functions.

### 3.3 mGluR5 Function in Depression

**FIGURE 8:**
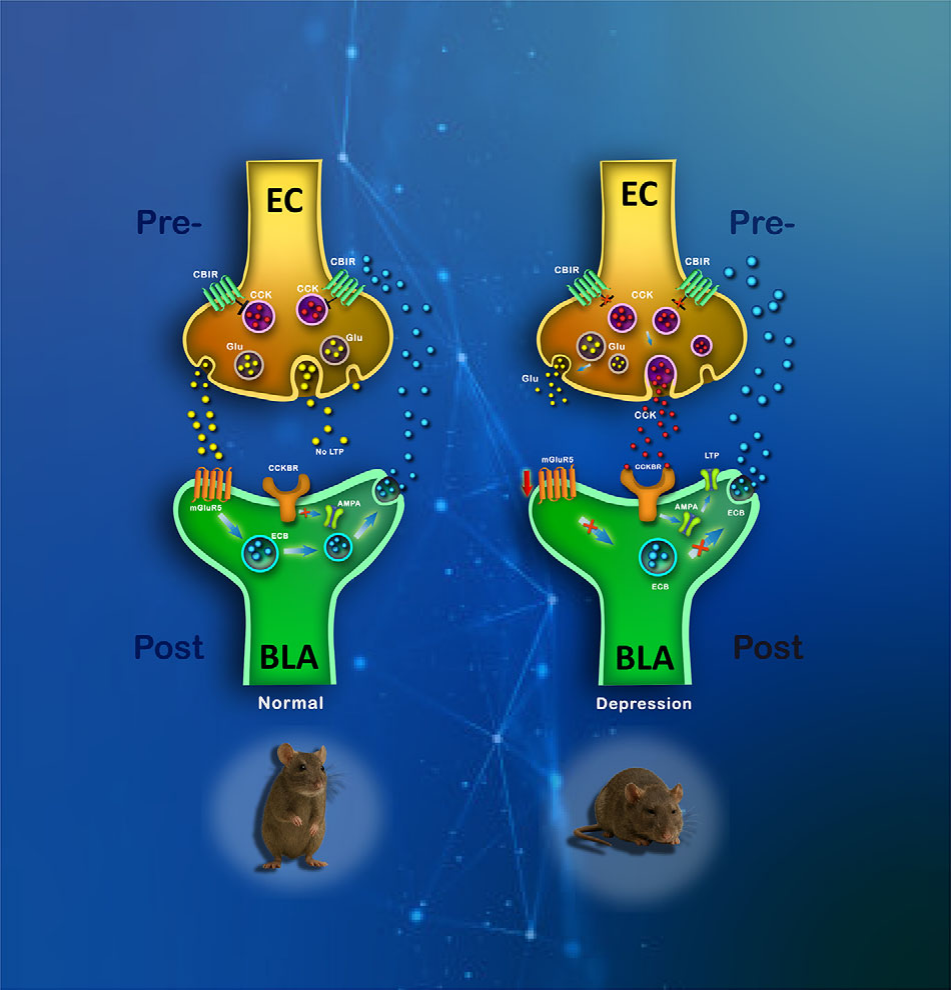
Proposed mechanism by which mGluR5 signaling in the EC^CCK^→BLA circuit regulates depressive‐like behavior.

Numerous studies have highlighted the significant role of mGluR5 in the pathogenesis of MDD [39, 40, 41]. Consistent with these findings, our study revealed that CSDS led to a decrease in mGluR5 levels, accompanied by an increase in depressive‐like phenotypes. Interestingly, we observed that mGluR5 located postsynaptically to EC^CCK^ to BLA pathways and regulated CCK release in this circuit through retrograde signaling to presynaptic CB1R. CB1Rs are well‐characterized components of CCK‐positive presynaptic terminals, [34, 49, 56] and our unpublished data confirm their expression on EC^CCK^ terminals in the BLA. Given that mGluR5 activation can initiate eCB release leading to CB1R‐mediated presynaptic inhibition [46, 47, 48], the observed downregulation of mGluR5 following CSDS may impair this retrograde signaling mechanism. Such disruption could result in disinhibited CCK release from EC^CCK^ terminals, promoting long‐term potentiation (LTP) in the BLA and facilitating the emergence of depressive‐like behaviors (Figure 8). A previous study also reported that mGluR5 agonists inhibit CCK release [38], supporting our current findings regarding the inhibitory effect of mGluR5 agonists on CCK release and their potential to alleviate depressive‐like symptoms. Moreover, we found that the induction of depressive‐like phenotypes by mGluR5 antagonists was dependent on the presence of CCK, as the antagonist failed to induce depressive‐like phenotypes in the absence of CCK, while in the presence of CCK4, mGluR5 antagonist facilitated depression. Discrepancies among previous studies have been observed, with some showing that antagonizing or decreasing mGluR5 levels are associated with increased depressive‐like phenotypes [39, 40, 41, 42, 44, 80], while others have demonstrated that loss of mGluR5 function promotes anti‐depressive behavior [81, 82]. These inconsistencies may arise from the diverse roles of mGluR5 in different brain regions or from variations in animal models and stress protocols. For example, loss of mGluR5 function under mild or acute stress protocols may facilitate anti‐depressive phenotypes [81, 82], whereas strong conditioning or chronic stress may trigger depressive phenotypes [43, 44]. It would be intriguing to investigate how varying degrees of stress affect CCK signaling and release in different brain regions and their correlation with mGluR5. Previous studies have demonstrated that mGluR5 inhibits presynaptic release through endocannabinoid‐CB1R signaling [46, 47], and CB1R are present on presynaptic glutamatergic [83] and CCK+ glutamatergic terminals [34]. Therefore, another potential mechanism is that reduced mGluR5 levels following CSDS lead to decreased endocannabinoid signaling and Cb1‐mediated presynaptic inhibition of CCK release in the EC^CCK^→BLA circuit, thereby triggering depressive‐like phenotypes in mice.

### 3.4 Synaptic Plasticity and Depression

Memories are formed in neuronal networks through two forms of synaptic plasticity: long‐term potentiation (LTP) and long‐term depression (LTD), which are recognized as crucial mechanisms underlying memory formation [84]. The precise relationship between synaptic plasticity and specific behaviors, however, remains a topic of debate [85, 86]. Nonetheless, synaptic plasticity is thought to play a role in depression, as alterations in synaptic plasticity have been associated with depression‐like phenotypes [87, 88]. Our recent studies have shown that CCK facilitates synaptic plasticity in the BLA and that blocking the CCKBR not only impairs the formation of LTP but also alleviates depressive‐like phenotypes in mice [31, 37]. In our current investigation, we demonstrated that activating mGluR5 impairs LTP in the BLA. However, when CCK4 was administered, it blocked the effect of mGluR5 activation. Given that mGluR5 receptors are co‐localized with CCK terminals in the BLA and regulate CCK release, and considering the crucial role of CCK in LTP formation in the BLA [31, 37], we conclude that mGluR5, through CCK signaling, modulates synaptic plasticity and depressive‐like phenotypes. While it has been reported that fear memories can be activated and deactivated by LTP and LTD in the BLA [89], it is worth noting that modulation of synaptic plasticity may be a hallmark of the antidepressant effects [8, 11]. A key limitation of the present study is that we did not investigate the molecular adaptations downstream of EC^CCK^→BLA circuit activation or inhibition, nor did we identify the additional downstream molecular triggers linking this circuit to depressive‐like behavior. Future studies employing transcriptomic profiling, gene expression validation, and other molecular approaches will be essential to delineate the downstream signaling pathways and cellular mechanisms that mediate these effects. Moreover, future studies should investigate the role of EC^CCK^ to BLA circuit across additional models, such as the chronic unpredictable mild stress (UCMS) model, to further strengthen the evidence that this circuit is critical for mediating stress‐induced depressive disorders.

Under physiological conditions, glutamate release from EC^CCK^ neurons activates postsynaptic mGluR5 receptors in the BLA, triggering retrograde endocannabinoid signaling. The released endocannabinoids bind to presynaptic CB1 receptors on EC^CCK^ terminals, thereby suppressing CCK release and limiting LTP induction. In contrast, chronic stress reduces mGluR5 expression, impairing endocannabinoid signaling. This disinhibits CCK release, leading to enhanced activation of postsynaptic CCKB receptors, increased AMPA receptor trafficking, and facilitation of synaptic potentiation, ultimately contributing to depressive‐like phenotypes.

## 4 Method and Materials

### 4.1 Animals

All experimental procedures conducted in this study were in accordance with the ethical guidelines established by the Animal Advisory Committee at the City University of Hong Kong for the Care and Use of Laboratory Animals (Reference # A307 & AN‐STA‐00000278). Approval was obtained prior to the commencement of any experiments. For the behavioral tests, immunohistochemistry, fiber photometry, optogenetics, and anatomy experiments, male C57BL/6 J mice aged 8–12 weeks, CCK‐ires‐Cre mice (Ccktm1.1(Cre)Zjh/J, C57BL/6 J background, stock # 012706, Jackson Laboratory) were utilized. The CCK‐CreER mouse line (Jackson Laboratory, Strain #012710; also referred to as CCK‐CreER‐KI/KO or CCK‐CreERT2‐KI/KO) was a knock‐in allele in which the endogenous cholecystokinin (CCK) gene was disrupted and replaced with CreERT2. This design abolishes CCK gene function and expression while enabling tamoxifen‐inducible Cre recombination in CCK‐expressing neurons (including both interneurons and pyramidal neurons), under the control of the endogenous CCK promoter/enhancer elements. The mice were housed in social groups of five per cage and maintained in a typical housing environment. The cages were furnished with corn cob litter, and the animals were kept in a temperature‐controlled animal room maintained at 23 ± 1 °C. A 12‐hour light/dark cycle was implemented, with lights on from 8:00 to 20:00 daily. The mice had access to food and water ad libitum. To ensure consistency, all behavioral experiments were conducted during the night time period.

### 4.2 Viruses

The following viruses were utilized in this study: AAV9‐CAMKII‐GCAMP6s (viral titer: 2.50 × 10^13^), AAV‐Retro‐EF1a‐DIO‐EYFP (viral titer: 1.30 × 10^13^), AAV1‐hSyn‐Cre (viral titer: 1.90 × 10^13^), and AAV9‐Syn‐Flex‐Chronos‐GFP (viral titer: 1.30 × 10^13^), which were obtained from Addgene (MA, USA). Additionally, AAV9‐EF1a‐DIO‐EYFP (viral titer: 5.24 × 10^12^), AAV9‐hSyn‐CCK2.3 (viral titer: 3.50 × 10^13^), and AAV‐CAMKII‐DIO‐mCherry (viral titer: 3.09 × 10^12^) were purchased from BrainVTA company (Wuhan, China). Furthermore, AAV9‐mCamKII‐DIO‐ChrimsonR‐mCherry (viral titer: 1.26 × 10^13^), AAV9‐hSyn‐DIO‐eArchT3.0‐EGFP (viral titer: 1.02 × 10^13^), and AAV9‐hSyn‐DIO‐EGFP (viral titer: 2.13 × 10^13^) were obtained from Shanghai Taitool Bioscience, China. Viruses with titers of ≈10^1^ ^3^ GC/mL were diluted twofold with 5% glycerol prior to injection. An exception was made for AAV‐retro‐EF1α‐DIO‐EYFP, which was used at the original titer, as retrograde AAVs was not effective at lower titers based on our prior experience. Viral constructs used in this study have been validated in our previous work.

### 4.3 Virus Injection, Optical Fiber, and Cannula Implantation

For the bilateral stereotaxic injection of viruses into the basolateral amygdala (BLA) and entorhinal cortex (EC), mice were anesthetized with sodium pentobarbital (50 mg kg–1, i.p. injection). The injection coordinates were as follows: BLA (AP = −1.60 mm, ML = ±3.37 mm, DV = −4.00 mm from bregma and dura matter) and EC (AP = −4.20 mm, ML = ±3.85 mm, DV = −2.70 mm) based on the mouse brain atlas. Each side of the brain was infused with 300 nL of the virus at a rate of 50 nL/min. Following each injection, the needle was left in place for an additional 10 min before being gradually withdrawn.

To record the GCAMP signal using fiber photometry, mice were implanted with an optical fiber cannula (6 mm length, NA 0.37) held in a ceramic ferrule (Inper company) positioned over the BLA (AP: −1.60 mm, ML: ±3.37 mm, DV: −3.85 mm from bregma and dura matter). For optogenetic stimulation, mice were implanted with bilateral optical fiber cannulas (6 mm length, NA 0.37) held in ceramic ferrules (Inper company) over the BLA (AP: −1.60 mm, ML: ±3.37 mm, DV: −3.70 mm from bregma and dura matter). Additionally, for chemical manipulation, a steel cannula was implanted over the BLA (AP: −1.60 mm, ML: ±3.37 mm, DV: −3.85 mm).

### 4.4 Fiber Photometry‐Based Calcium Measurements

The fiber photometry recording procedure was conducted following the methodology described in our previous studies [10, 37]. Prior to each testing session, mice were attached with a fiber patch cord and allowed to habituate for a minimum of 10 min. Fluorescence signals were recorded using a fiber photometry system (Doric Lenses) equipped with two continuous sinusoidally modulated LEDs (DC4100, ThorLabs). The LEDs operated at 473 nm (220 Hz) and 405 nm (330 Hz) to excite GCaMP6s and an isosbestic autofluorescence signal, respectively. Both light sources were connected to a large‐core (200 µm), high‐NA (0.37) optical fiber patch cord, which was coupled to the corresponding brain implant in each mouse. The light intensity at the fiber tip interface was set to 15 µW. GCaMP6s and autofluorescence signals were captured by the same fiber and directed to separate photoreceivers (2151, Newport Corporation). An RZ5P acquisition system (Tucker‐Davis Technologies; TDT), equipped with a real‐time signal processor, controlled the LEDs and autonomously demodulated the fluorescence brightness resulting from the 473‐nm and 405‐nm excitation. Behavioral activities were recorded via TTL input using the same system.

During footshock‐induced activities, animals experienced mild footshocks (0.2 mA) delivered by a metal grid. The footshocks were administered three times, each lasting 2 s, with a 1‐minute interval between occurrences. For optogenetic laser activation of EC^CCK^ neurons, red light was applied to EC^CCK^ neurons for three occurrences, each lasting 2 s, with a 1‐minute interval between occurrences. CCK release was detected using a CCK sensor, as previously described [37]. Specifically, red light (40 Hz) was shone over EC^CCK^ neurons for three occurrences, each lasting 2 s, with a 1‐minute interval between occurrences, and CCK release was detected in the BLA using the CCK sensor. The acquired data were analyzed using pMAT, an open‐source software suite designed for the analysis of fiber photometry data [90]. The resulting fitted 405‐nm signal was utilized to normalize the 473‐nm signal through the following equation: ΔF/F0 = (473‐nm signal—fitted 405‐nm signal) / fitted 405‐nm signal.

### 4.5 Expansion Microscopy

After perfusion, mouse brains were isolated and immediately immersed in freshly prepared 4% paraformaldehyde (PFA) in phosphate‐buffered saline (PBS) for tissue fixation. The samples were incubated in PFA at 4°C overnight to ensure proper fixation. Following fixation, the paraformaldehyde‐treated brains were sectioned into 100 µm slices using a Leica Vibrating Blade Microtome. For pre‐immunostaining, the slices were washed three times with 1x PBS, with each wash lasting 10 min. After washing, the slices were blocked with 5% goat serum in PBST (0.3% Triton X‐100 in PBS) overnight. Subsequently, the blocked slices were incubated with primary antibodies (mGluR5, AB5675, Sigma‐Aldrich, diluted 1:100; PSD95, ab18258, Abcam, diluted 1:200) at 4°C for 48 h. After primary antibody staining, the slices were washed again with 1x PBS three times for 10 min each, followed by overnight incubation with secondary antibodies (CF‐633, 20120, Biotium, diluted 1:200; Alexa‐594 or 488, Thermo Fisher, diluted 1:200). For gelation and tissue embedding, a gelation solution was prepared by mixing 940 µL of Stock‐X comprising 225 µL sodium acrylate, 50 µL acrylamide, 75 µL N,N′‐methylenebisacrylamide, 400 µL sodium chloride, 100 µL 10x PBS stock, and 90 µL ddH2O with 20 µL of 10 g/100 mL tetramethylethylenediamine (TEMED), 20 µL of 10 g/100 mL ammonium persulfate (APS), and 20 µL of 0.5 g/100 mL 2,2,6,6‐tetramethylpiperidine‐1‐oxyl (TEMPO). Fixed tissues were then incubated in this gelation solution at 4°C for 1 h to facilitate thorough diffusion. Polymerization was initiated by incubating the samples at 37°C for 2 h in a humidified chamber. After polymerization, the tissues embedded in the hydrogel were washed with PBS to remove unreacted monomers. For protein digestion, a digestion buffer was prepared by dissolving Proteinase K (8 U/mL) in a buffer consisting of 50 mM Tris, 1 mM EDTA, 0.5% Triton X‐100, and 0.8 M NaCl at pH 8.0. The tissues were incubated in this digestion buffer at 37°C for 12 to 18 h, facilitating the expansion of the hydrogel by removing cross‐linked proteins within the tissue. After digestion, the tissues were washed with excess Milli‐Q water to induce swelling, with the water being replaced every 30 min for three cycles to achieve maximal expansion, approximately 4 to 5 times the original size. Finally, the expanded and stained tissue sections were imaged using a confocal laser scanning microscope (Nikon AXR) with 60x objective lens, allowing for detailed visualization of the samples. Imaging data were analyzed using Fiji ImageJ software.

### 4.6 In Vitro Electrophysiology

In vitro electrophysiological recording procedures were conducted following the methodology described in our previous study [37]. Artificial cerebrospinal fluid (ACSF) was prepared by dissolving 124 mM NaCl, 2.5 mM KCl, 1 mM NaH2PO4, 10 mM D‐glucose, 25 mM NaHCO3, 2 mM CaCl2, and 1 mM MgCl2 in distilled water. The pH of the ACSF was adjusted to 7.35–7.45. Before recording, the ACSF was well‐oxygenated (95% O2/5% CO2, v/v). Mice were deeply anesthetized with isoflurane to obtain brain slices. The brain was kept in oxygenated ice‐cold ACSF, and coronal sections of the amygdala with a thickness of 300 µm were prepared using a vibratome. The brain slices were then incubated in oxygenated ACSF at 30 ± 1°C for 1.5 h to allow for recovery. After recovery, a brain slice was placed on the microelectrode array system probe (MED‐PG515A) on the microscope stage. A fine mesh and anchor were carefully positioned on the slice to prevent floating during recording. The slices were continuously perfused with oxygenated ACSF at 30 ± 1°C. The microelectrode array was positioned on the targeted brain region under a light microscope, and the stimulation site was chosen based on microscope photos. Microelectrodes were screened, and the optimal stimulation site was selected. Following a 30‐minute recovery period with stable baseline field excitatory postsynaptic potential (fEPSP) responses, an input‐output curve was determined by measuring the amplitude of fEPSPs in response to a series of ascending stimulation intensities in 10 mA steps. For long‐term potentiation (LTP) induction, a stimulation intensity of 30%–50% saturation was selected for baseline synaptic response recording. The fEPSPs were stable for at least 15 min to establish a baseline. Subsequently, a theta burst stimulation (TBS) protocol consisting of 4 sets with 10‐second intervals was administered at 5 Hz, with each set containing 5 trains of 4 pulses at 100 Hz. The stimulation intensity for TBS was adjusted to elicit 75% of the saturated intensity to induce LTP.

For testing the effects of drugs, after the baseline recording, the ACSF was perfused with a mGluR5 agonist (DHPG) for 10 min prior to TBS. The fEPSP responses were recorded for at least 60 min after TBS. Changes in fEPSP amplitudes were expressed as a percentage change from the baseline. The average normalized amplitudes of the first 10 min before and the last 10 min after TBS were compared. To compare the magnitude of LTP between groups, the averaged values of the last 10 min were statistically compared. Slices with unstable baselines were excluded from the study.

### 4.7 Immunohistochemistry

After anesthetizing the mice with pentobarbital sodium overdose, they were transcardially perfused with 30 mL of phosphate‐buffered saline (PBS) followed by 30 mL of 4% paraformaldehyde (PFA) in PBS, 60 min after sensory stimuli. The brains were then removed and fixed with 4% PFA overnight. Coronal sections with a thickness of 50 µm were obtained using a vibratome. The brain sections were washed with PBS and blocked with 5% goat serum in PBST (0.3% Triton X‐100 in PBS) for 2 h. Subsequently, the slices were incubated with the primary antibody anti‐c‐fos rabbit (ab190289) at a concentration of 1:1000 for 24 h at 4°C. After washing three times with PBS, the slices were incubated with secondary antibodies, either Alexa Fluor 488 (1:500) or Alexa Fluor 647 (1:500), at room temperature for 2 h. Following another three washes with PBS, the slices were incubated with DAPI (1:5000) for 5 min and washed again three times with PBS. The slices were then mounted on slides, and images were captured using a Nikon confocal microscope. Data analysis was performed using Image J software.

### 4.8 Western Blotting

Mice were sacrificed following chronic social defeat stress (CSDS), and their brains were immediately isolated on ice. Coronal sections were prepared, and tissues from the BLA were collected in RIPA buffer containing a protease inhibitor (1:100). The samples were homogenized and centrifuged at 12,000 g for 20 min at 4°C. The supernatants were transferred to fresh tubes, and protein concentrations were determined using a BCA assay (Pierce, Waltham, MA, Catalog# 23225). Proteins were resolved by SDS‐PAGE and subsequently transferred to a PVDF membrane (Immobilon‐P, Millipore). The membranes were incubated overnight at 4°C with an anti‐mGLUR5 antibody (Catalog# AB5675; abcam). Membranes were washed three times with TBST for 10 min each and then incubated with an HRP‐conjugated secondary antibody (1:1000, Catalog# HAF008, R&D, MN). Protein signals were developed using Immobilon Western Chemiluminescent HRP Substrate (Millipore). The immunoreactivity of the target protein was normalized to GAPDH (1:1000, Catalog# ab8245, abcam) and quantified by densitometry using ImageJ (NIH, MD).

### 4.9 Optogenetic Alleviation or Induction of Depressive‐Like Behavior

To investigate the effects of optogenetic manipulation on depressive‐like behavior, mice were injected with either AAV9‐hSyn‐DIO‐eArchT3.0‐EGFP or AAV9‐hSyn‐DIO‐EGFP in the EC of CCK‐Cre mice. For optogenetic inhibition of the EC→BLA circuit, mice were connected to an optical patch cord and placed on opposite sides of CD‐1 aggressor mice using a divider. Yellow light inhibition was delivered for 4 s on and 1 s off for 10 min, repeated for three consecutive days following chronic social defeat stress.

Similarly, mice injected with either AAV9‐Syn‐Flex‐Chronos‐GFP or AAV9‐EF1a‐DIO‐EYFP in the EC of CCK‐Cre mice were used for optogenetic activation of the EC→BLA circuit. Mice were attached to an optical patch cord and placed on opposite sides of CD‐1 aggressor mice using a divider. Blue light activation was administered for 0.125 s on and 0.875 s off for 10 min following subthreshold social defeat.

### 4.10 Chemical Alleviation or Induction of Depressive‐Like Behavior

To examine the impact of chemical activation or inhibition on depressive‐like behavior, mGluR5 in the basolateral amygdala (BLA) was targeted. For chemical activation of mGluR5, mice received infusions of 3,5‐dihydroxyphenylalanine (DHPG) (100 µM, 0.5 µl) at a rate of 100 nL/min for ten consecutive days, with infusions performed 30 min before each trial of social defeat stress. In contrast, for chemical inhibition of mGluR5, mice received infusions of 2‐methyl‐6‐(phenylethynyl) pyridine hydrochloride (MPEP) (100 µM, 0.5 µl) at a rate of 100 nL/min immediately after the subthreshold social defeat.

### 4.11 Behavior Assays

#### 4.11.1 Real‐Time Place Aversion (RTPA)

Real‐time place aversion (RTPA) was performed three weeks after surgery on mice expressing AAV9‐mCamKII‐DIO‐ChrimsonR‐mCherry and their control counterparts expressing AAV‐CAMKII‐DIO‐mCherry. Optical fibers were bilaterally implanted above the basolateral amygdala (BLA). The mice were placed in a behavioral arena (32 cm × 32 cm) consisting of two chambers and habituated to the environment for 10 min before the testing session. One side of the chamber was designated as the stimulation site. During the 10‐minute test, the mouse was initially placed on the non‐stimulated side, and each time it crossed to the stimulation site, 40 Hz laser stimulation was delivered until the mouse returned to the non‐stimulation side. The movements of the mice were tracked using a video camera positioned above the chamber, and Smart 3 software was used for analysis. The avoidance score was calculated as (Time in Laser ON Side ÷ Total Time) × 100.

#### 4.11.2 Chronic Social Defeat Stress

Chronic social defeat stress (CSDS) was conducted following established protocols [91]. Retired male breeder CD1 mice were assessed for their aggressive traits over three consecutive days before the experiment. The experimental male C57BL6/J or CCK‐Cre mouse (intruder) was then placed in the home cage of a novel aggressive CD1 mouse (resident) for 5–10 min of physical defeat, repeated for 10 consecutive days. Following the physical defeat, the experimental mice were maintained in sensory contact for 24 h by being placed on the opposite side of the CD1 aggressor using a perforated Plexiglass partition. After CSDS, the mice were housed individually, and a social interaction test was conducted 24 h later. Control mice were paired on opposite sides of the perforated Plexiglass partition in the same cage for 10 days without any physical defeat.

#### 4.11.3 Social Interaction

Social avoidance behavior was assessed using a two‐trial social interaction test. In the first trial, mice were introduced into an open‐field chamber (44 × 44 × 44 cm3) containing an empty cage made of mesh wire and plastic sheets (10 × 6 × 8 cm3). The mice were tracked using Debut video‐tracking software. The time spent in the social interaction zone (8 cm around the cage) was quantified individually. This first trial was referred to as ‘No target.’ After a one‐minute period in the home cage, a novel CD1 aggressor mouse was introduced into the cage for the second trial, referred to as ‘Target.’ The social interaction ratio was evaluated as (time spent in the interaction zone with a Target/time spent in the interaction zone with No target).

#### 4.11.4 Two‐Trial Subthreshold Social Defeat Stress

A two‐trial subthreshold social defeat (SSDS) stress experiment was performed following established protocols [10, 45]. The experimental mouse was placed in the home cage of an aggressive CD1 mouse for 10 min of physical defeat. After 10 min of physical stress, the experimental mice underwent 10 min of sensory stimuli on the opposite side of a CD1 mouse using a perforated Plexiglass wall. Subsequently, the mice experienced a second trial of physical defeat with a novel CD1 mouse. The behavior tests to evaluate depressive‐like behavior were conducted on the following days.

#### 4.11.5 Sucrose Preference Test

Following the social interaction test, mice were habituated for two consecutive days to 50 mL tubes with rubber stoppers and ballpoint steel sipper tubes (two‐bottle choice). One of the bottles was then replaced with 1% sucrose to determine sucrose preference. The position of the bottles was changed at specific intervals to ensure no side preference was developed. Sucrose preference was calculated as the percentage (amount of sucrose consumed × 100 (bottle a)/total volume consumed (bottles a + b)). Total sucrose intake over 24 h was quantified to determine sucrose preference.

#### 4.11.6 Tail‐Suspension Test

Mice were suspended by their tails using adhesive tape positioned 1 cm from the tail tip, approximately 50 cm above the surface to prevent contact. Plastic tubes were placed on the tails to prevent climbing or hanging. The mice were recorded with a video camera for 6 min, and the duration of immobility during this period was used to determine despair‐like behavior.

#### 4.11.7 Open‐Field Test

Mice were individually placed in the central zone of an open‐field arena (44 × 44 cm^2^) with dim light during the dark phase for 10 min. Their movements were recorded by a video camera and analyzed using Smart 3.0 software. Anxiety‐like behavior was assessed based on the time spent in the central zone.

### 4.12 Quantification and Statistical Analyses

All data were presented as means ± s.e.m. Statistical analyses were performed using GraphPad Prism 8.0.1 software with appropriate inferential methods, as specified in Table S1. Paired and unpaired t‐tests were used for two‐group comparisons of normally distributed data, While Mann‐Whitney U test were performed for data that were not distributed normally. One‐ and two‐way analysis of variance (ANOVA) was used for multiple group comparisons, followed by the Bonferroni test for multiple comparisons. Statistical significance was set at *P < 0.05, **P < 0.01, ***p < 0.001, and ****P < 0.0001.

## Author Contributions

**J.H**. and **M.A**. designed the experiments; **M.A**. collected and analyzed the data for fiber photometry; **M.A. and H.W**. collected and analyzed the data for the behavioral part. **H.W**., **G.Q**., and **A.W**. performed immunohistochemistry. **M.A**. wrote the original draft of the manuscript.

## Conflicts of Interest

The authors declare no conflicts of interest.

## Supporting information




**Supporting File**: advs75615‐sup‐0001‐SuppMat.docx.

**Supporting file**: advs75615‐sup‐0002‐Table S1_production.xlsx.

## Data Availability

The data that support the findings of this study are available from the corresponding author upon reasonable request.
