# Supplementary material for: mGluR5 in ECCCK to BLA Circuit Modulates Depressive‐Like Phenotypes through CCK Signaling
Source: Adv Sci (Weinh). 2026 May 8;13(43):e23115. doi: 10.1002/advs.202523115 (PMC13335908; doi:10.1002/advs.202523115)
Supplement: Supplementary file 1 — Supporting File 1: advs75615‐sup‐0001‐SuppMat.docx. [file ADVS-13-e23115-s001.docx]

**Supporting Information**

**Title:** mGluR5 in EC^CCK^ to BLA Circuit Modulates Depressive-like Phenotypes Through CCK Signaling.

# Authors: Muhammad Asim^1,2,4,5*^, Huajie Wang^1,5^, Gao Qianqian^1^, Abdul Waris^1^, Jufang He^1,2,3*^

**
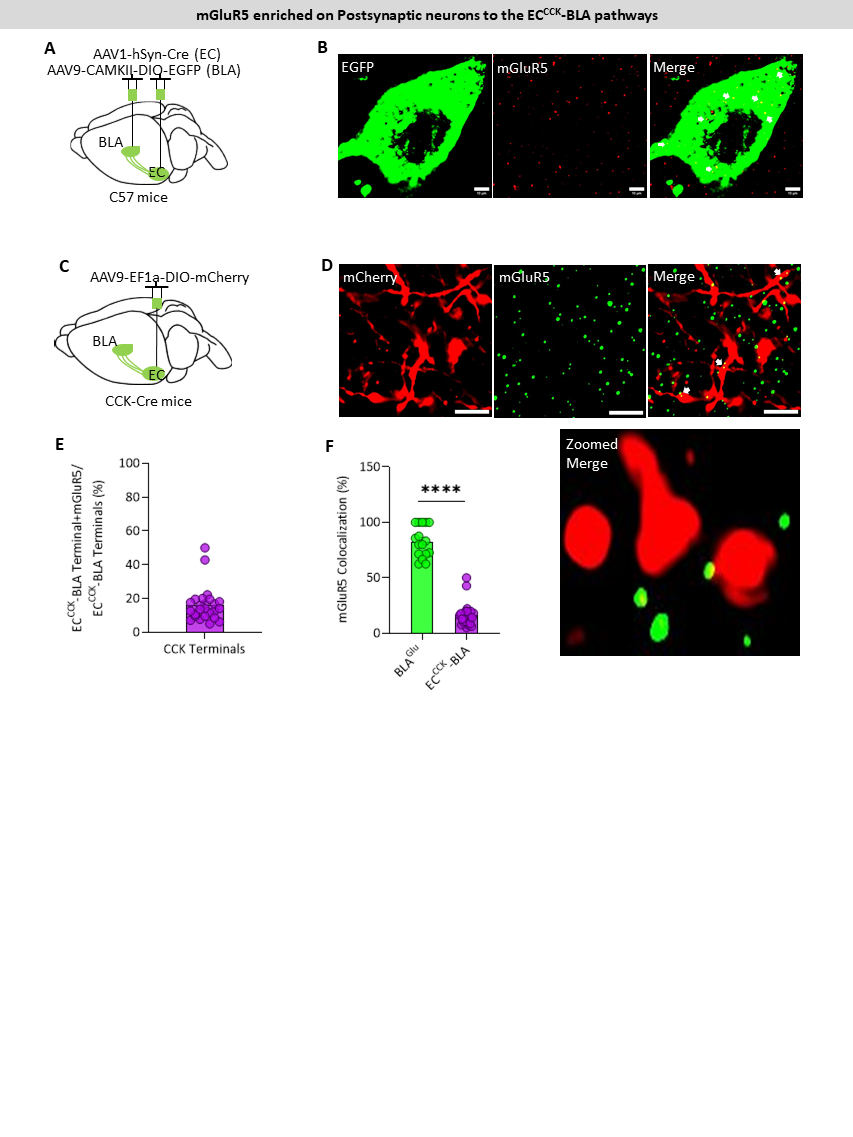
**

# *Figure S1: mGluR5 enriched on Postsynaptic neurons to the EC^CCK^-BLA pathways.*

**A**. Schematic design for virus injection. **B.** Image showing the colocalization of mGLUR5 with BLA^Glu^ neurons (cell-body) which receive input from EC. Left: BLA^Glu^ neuron (Green), mGluR5 (red), Yellow (merge), Scale bar 10 um. **C.** Schematic design for virus injection in EC of CCK-Cre mice. **D.** Image showing the colocalization of mGLUR5 with EC^CCK^ terminal in the BLA. Left: EC^CCK^ terminals in BLA (Red), mGluR5 (Green), Yellow (merge), Scale bar 10 um; Bottom right: a zoomed image. **E.** Average percentage of colocalized terminals with mGLUR5. **F.** Comparison of average percentage colocalized BLA^Glu^ terminals and EC^CCK^ terminals in BLA.

All data are shown as mean ± s.e.m. *P < 0.05; **P < 0.01; ***P < 0.001; ****P < 0.0001 (see Table S1 for detailed statistics).


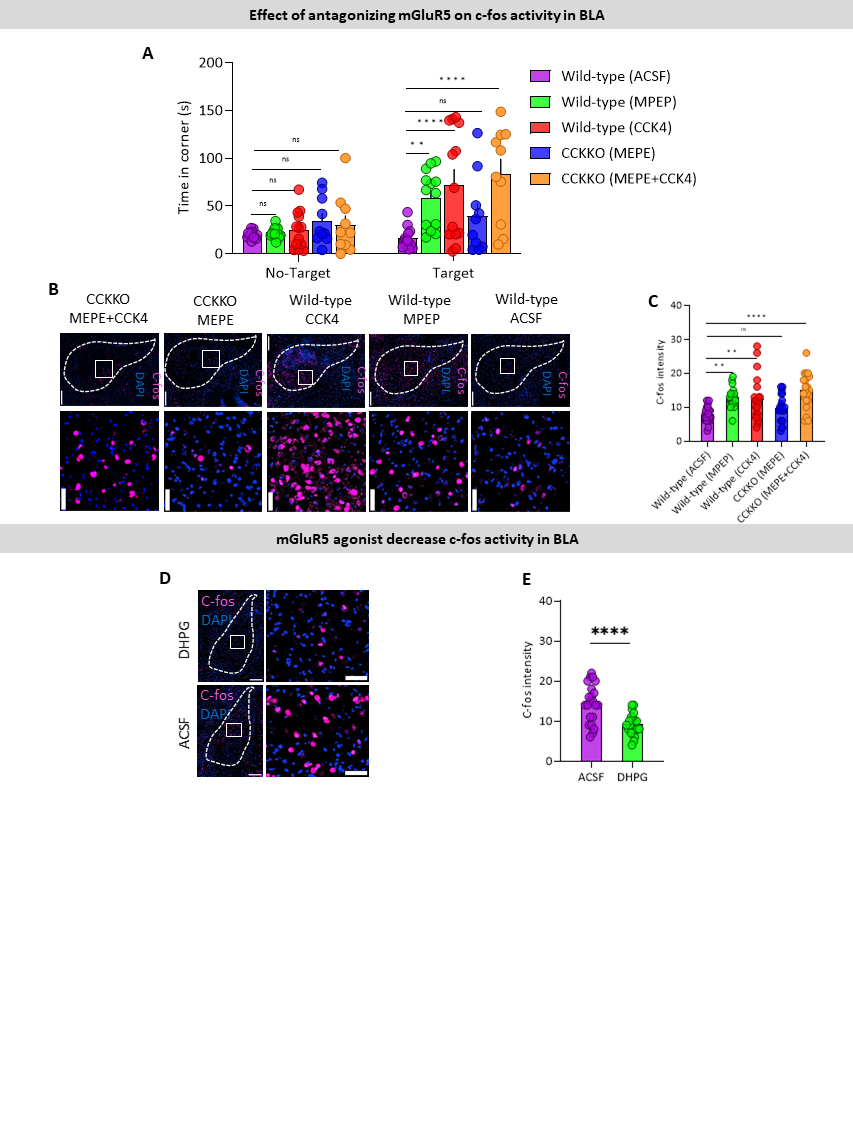


# *Figure S2: mGluR5 effect on c-fos activity in the BLA.*

**A.** Time spent in a corner among Wildtype ACSF, Wildtype MPEP, Wildtype CCK4, CCKKO (MPEP), and CCKKO (MPEP+CCK4) in the presence of Target or No-target during SIT. **B.** Representative image of c-fos expression; Top: enlarged 200um; Bottom: zoomed 50um. **C.** Average c-fos intensity among Wildtype ACSF, Wildtype MPEP, Wildtype CCK4, CCKKO (MPEP), and CCKKO (MPEP+CCK4) groups. **D.** Representative image of c-fos expression; Left: enlarged 200um; right: zoomed 50um. **E.** Average c-fos intensity among ACSF vs. DHPG group.

All data are shown as mean ± s.e.m. *P < 0.05; **P < 0.01; ***P < 0.001; ****P < 0.0001 (see Table S1 for detailed statistics).


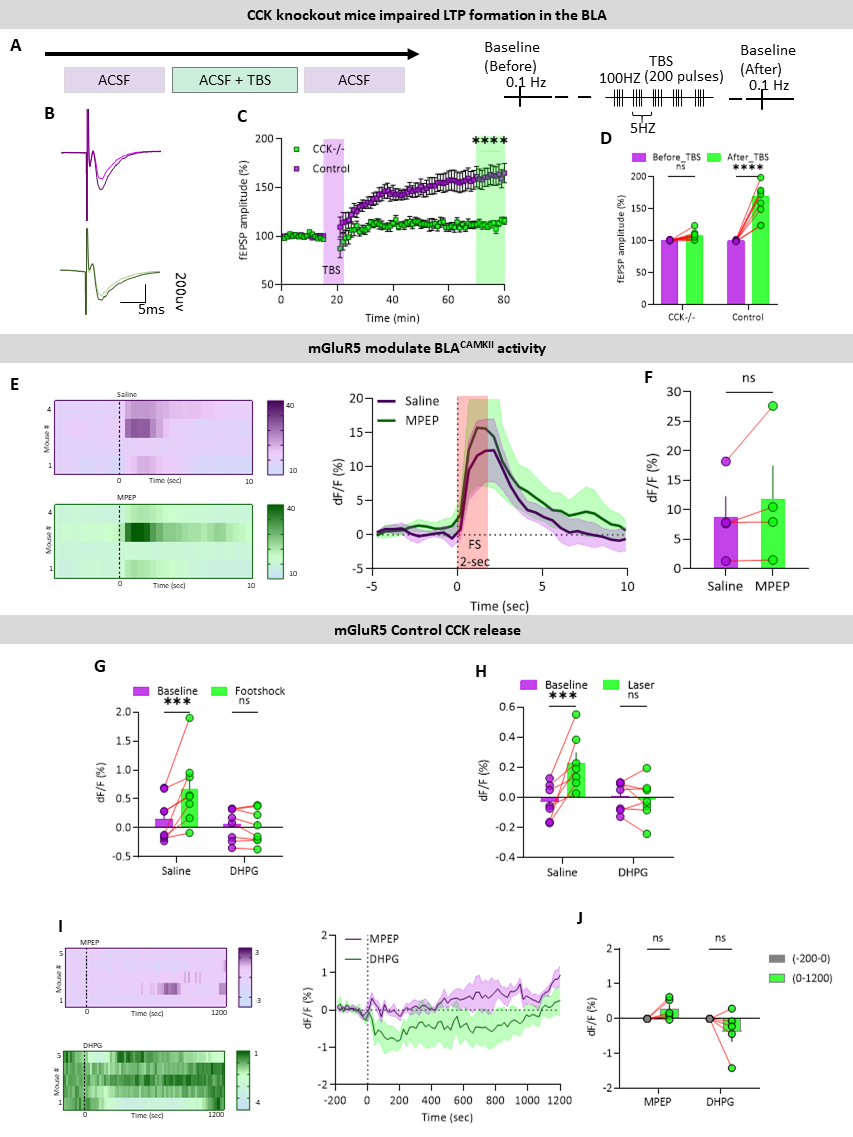


# *Figure S3: mGluR5 control CCK release in EC^CCK^ to the BLA circuit.*

**A.** Left: Schematic representation of the experimental design. Right: protocol for baseline and TBS. **B.** Representative of single field excitatory postsynaptic potential (fEPSP) before and after TBS in control and CCK-/-. **C.** % fEPSPs amplitude traces before and after TBS in a control (purple) and CCK-/- (Green). **D.** Average change in fEPSPs (%) before and after TBS in control and CCK-/-. **E.** Left: Heat map; Right: Average calcium traces before and during footshock. **F.** Individual responses among saline and MPEP groups. **G.** Individual responses among baseline and footshock saline and DHPG groups. **H.** Individual responses during baseline and laser activation of EC^CCK^ neurons among saline and DHPG groups. **I.** Left: Heat map; Right: Average CCK traces before and after Injection of MPEP or DHPG. **J.** Average and individual change in CCK sensor during Baseline vs. after DHPG injection, or Baseline vs. after MPEP injection.

All data are shown as mean ± s.e.m. *P < 0.05; **P < 0.01; ***P < 0.001; ****P < 0.0001 (see Table S1 for detailed statistics).


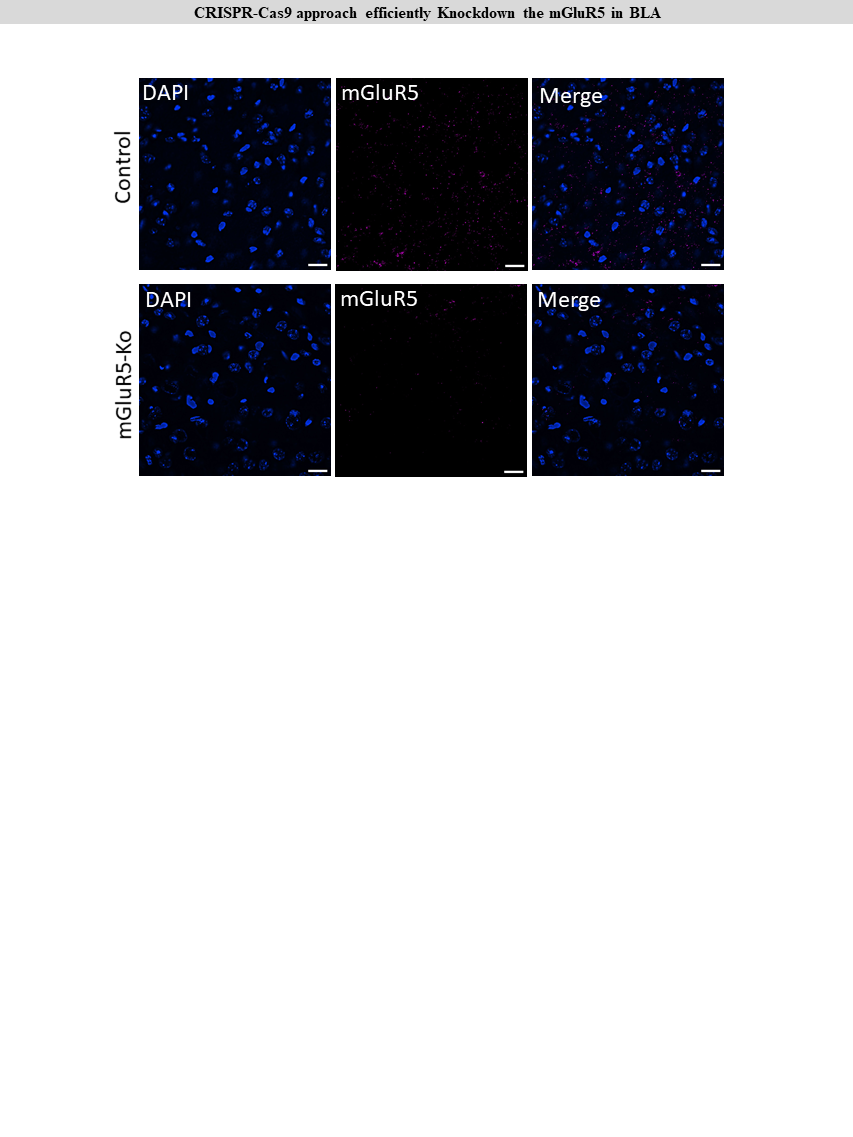


***Figure S4. CRISPR–Cas9–mediated knockdown of mGluR5 in the BLA.***
Top row: Representative images from control mice showing DAPI (left), mGluR5 (middle), and merged signals (right); scale bar = 20 μm. Bottom row: Representative images following CRISPR–Cas9–mediated knockdown showing DAPI (left), mGluR5 (middle), and merged signals (right); scale bar = 20 μm.


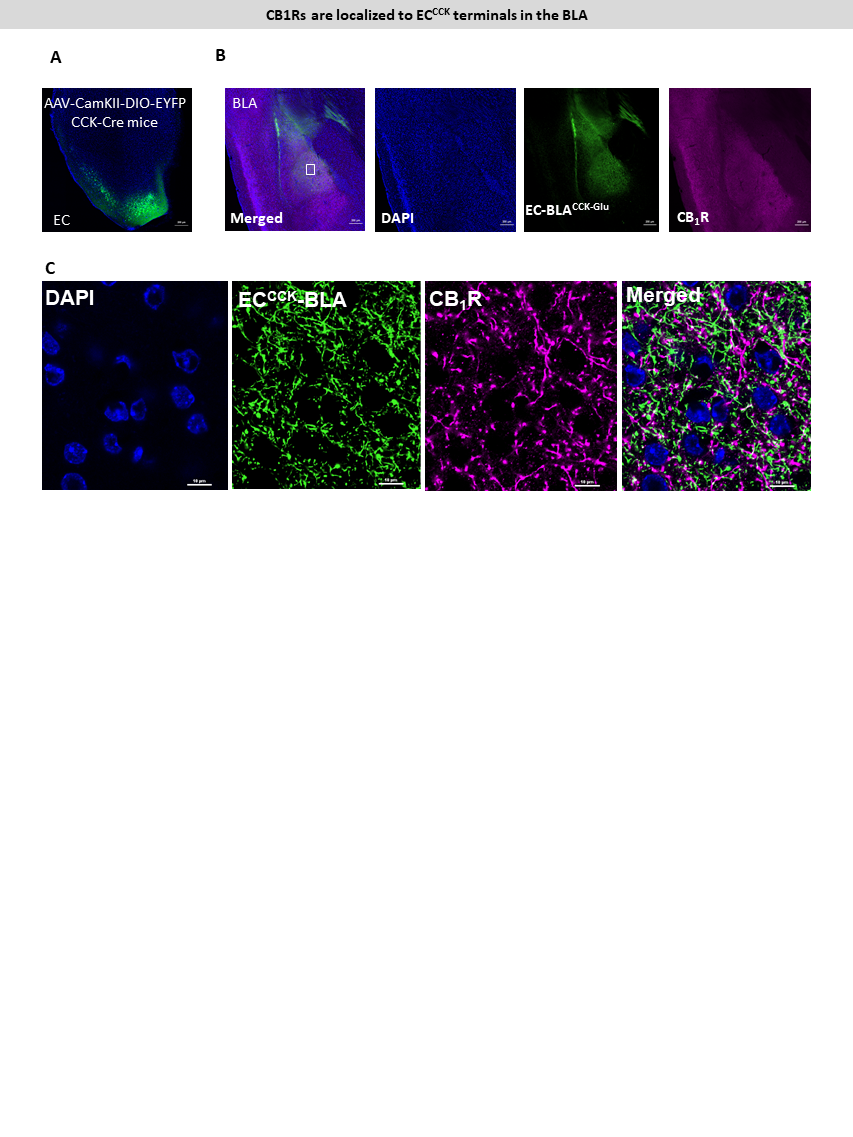


# *Figure S5. Anatomical localization of CB1R at EC^CCK^→BLA presynaptic terminals.* A. Viral expression in the EC. B. EC^CCK^ axon terminals within the BLA. C. High-magnification images demonstrating colocalization of CB1R with EC^CCK^ terminals in the BLA.
